# Assessing the role of insulin‐like growth factors and binding proteins in prostate cancer using Mendelian randomization: Genetic variants as instruments for circulating levels

**DOI:** 10.1002/ijc.30206

**Published:** 2016-06-23

**Authors:** Carolina Bonilla, Sarah J. Lewis, Mari‐Anne Rowlands, Tom R. Gaunt, George Davey Smith, David Gunnell, Tom Palmer, Jenny L. Donovan, Freddie C. Hamdy, David E. Neal, Rosalind Eeles, Doug Easton, Zsofia Kote‐Jarai, Ali Amin Al Olama, Sara Benlloch, Kenneth Muir, Graham G. Giles, Fredrik Wiklund, Henrik Grönberg, Christopher A. Haiman, Johanna Schleutker, Børge G. Nordestgaard, Ruth C. Travis, Nora Pashayan, Kay‐Tee Khaw, Janet L. Stanford, William J. Blot, Stephen Thibodeau, Christiane Maier, Adam S Kibel, Cezary Cybulski, Lisa Cannon‐Albright, Hermann Brenner, Jong Park, Radka Kaneva, Jyotsna Batra, Manuel R. Teixeira, Hardev Pandha, the PRACTICAL consortium, Mark Lathrop, Richard M. Martin, Jeff M. P. Holly

**Affiliations:** ^1^ School of Social and Community MedicineUniversity of BristolBristolUnited Kingdom; ^2^ MRC/University of Bristol Integrative Epidemiology Unit, University of BristolBristolUnited Kingdom; ^3^ Department of Mathematics and StatisticsLancaster UniversityLancasterUnited Kingdom; ^4^ Nuffield Department of SurgeryUniversity of OxfordOxfordUnited Kingdom; ^5^ Surgical Oncology (Uro‐Oncology: S4), University of CambridgeBox 279, Addenbrooke's Hospital, Hills RoadCambridgeUnited Kingdom; ^6^ The Institute of Cancer Research15 Cotswold RoadSuttonSurreySM2 5NGUnited Kingdom; ^7^ Royal Marsden NHS Foundation Trust, Fulham and Sutton, London and SurreyUnited Kingdom; ^8^ Centre for Cancer Genetic Epidemiology, Department of Public Health and Primary CareUniversity of Cambridge, Strangeways Research LaboratoryWorts CausewayCambridgeUnited Kingdom; ^9^ University of WarwickCoventryUnited Kingdom; ^10^ Institute of Population Health, University of ManchesterManchesterM13 9PLUnited Kingdom; ^11^ The Cancer Council Victoria615 St. Kilda RoadMelbourneVictoria3004Australia; ^12^ Centre for Epidemiology and Biostatistics, Melbourne School of Population and Global Healththe University of MelbourneVictoria3010Australia; ^13^ Department of Medical Epidemiology and BiostatisticsKarolinska InstituteStockholmSweden; ^14^ Department of Preventive Medicine, Keck School of MedicineUniversity of Southern California/Norris Comprehensive Cancer CenterLos AngelesCalifornia; ^15^ Department of Medical Biochemistry and GeneticsUniversity of TurkuTurkuFinland; ^16^ Institute of Biomedical Technology/BioMediTech, University of Tampere and FimLab LaboratoriesTampereFinland; ^17^ Department of Clinical BiochemistryHerlev Hospital, Copenhagen University HospitalHerlev Ringvej 75HerlevDK2730Denmark; ^18^ Cancer Epidemiology Unit, Nuffield Department of Population HealthUniversity of OxfordOxfordUnited Kingdom; ^19^ Centre for Cancer Genetic Epidemiology, Department of OncologyUniversity of Cambridge, Strangeways Research LaboratoryWorts CausewayCambridgeUnited Kingdom; ^20^ Department of Applied Health ResearchUniversity College London1‐19 Torrington PlaceLondonWC1E 7HBUnited Kingdom; ^21^ Forvie SiteCambridge Institute of Public Health, University of CambridgeRobinson WayCambridgeCB2 0SRUnited Kingdom; ^22^ Division of Public Health SciencesFred Hutchinson Cancer Research CenterSeattleWashington; ^23^ Department of Epidemiology, School of Public HealthUniversity of WashingtonSeattleWashington; ^24^ International Epidemiology Institute1455 Research Blvd, Suite 550RockvilleMaryland; ^25^ Mayo ClinicRochesterMinnesota; ^26^ Department of UrologyUniversity Hospital UlmGermany; ^27^ Institute of Human Genetics, University Hospital UlmGermany; ^28^ Brigham and Women's Hospital/Dana‐Farber Cancer Institute45 Francis Street‐ASB II‐3BostonMassachussets; ^29^ Washington UniversitySt LouisMissouri; ^30^ International Hereditary Cancer Center, Department of Genetics and PathologyPomeranian Medical UniversitySzczecinPoland; ^31^ Division of Genetic Epidemiology, Department of MedicineUniversity of Utah School of MedicineSalt Lake CityUtah; ^32^ Division of Clinical Epidemiology and Aging ResearchGerman Cancer Research Center (DKFZ)HeidelbergGermany; ^33^ Division of Preventive OncologyGerman Cancer Research Center (DKFZ)HeidelbergGermany; ^34^ German Cancer Consortium (DKTK), German Cancer Research Center (DKFZ)HeidelbergGermany; ^35^ Division of Cancer Prevention and ControlH. Lee Moffitt Cancer Center12902 Magnolia DrTampaFlorida; ^36^ Molecular Medicine Center and Department of Medical Chemistry and BiochemistryMedical University ‐ Sofia2 Zdrave StSofia1431Bulgaria; ^37^ Australian Prostate Cancer Research Centre‐Qld, Institute of Health and Biomedical Innovation and School of Biomedical SciencesQueensland University of TechnologyBrisbaneAustralia; ^38^ Department of GeneticsPortuguese Oncology InstitutePortoPortugal; ^39^ Biomedical Sciences Institute (ICBAS), Porto UniversityPortoPortugal; ^40^ The University of SurreyGuildfordSurreyGU2 7XHUnited Kingdom; ^41^ Commissariat à L'Energie Atomique, Center National De GénotypageEvryFrance; ^42^ McGill University‐Génome Québec Innovation CentreMontrealCanada; ^43^ NIHR Bristol Biomedical Research Unit in NutritionBristolUnited Kingdom; ^44^ IGFs and Metabolic Endocrinology Group, School of Clinical Sciences North BristolUniversity of BristolBristolUnited Kingdom

**Keywords:** insulin‐like growth factors, insulin‐like growth factor‐binding proteins, prostate cancer, Mendelian randomization, single nucleotide polymorphisms, IGFBP3, ProtecT, PRACTICAL, ALSPAC, UKHLS

## Abstract

Circulating insulin‐like growth factors (IGFs) and their binding proteins (IGFBPs) are associated with prostate cancer. Using genetic variants as instruments for IGF peptides, we investigated whether these associations are likely to be causal. We identified from the literature 56 single nucleotide polymorphisms (SNPs) in the IGF axis previously associated with biomarker levels (8 from a genome‐wide association study [GWAS] and 48 in reported candidate genes). In ∼700 men without prostate cancer and two replication cohorts (*N* ∼ 900 and ∼9,000), we examined the properties of these SNPS as instrumental variables (IVs) for IGF‐I, IGF‐II, IGFBP‐2 and IGFBP‐3. Those confirmed as strong IVs were tested for association with prostate cancer risk, low (< 7) *vs*. high (≥ 7) Gleason grade, localised *vs*. advanced stage, and mortality, in 22,936 controls and 22,992 cases. IV analysis was used in an attempt to estimate the causal effect of circulating IGF peptides on prostate cancer. Published SNPs in the *IGFBP1/IGFBP3* gene region, particularly rs11977526, were strong instruments for IGF‐II and IGFBP‐3, less so for IGF‐I. Rs11977526 was associated with high (*vs*. low) Gleason grade (OR per IGF‐II/IGFBP‐3 level‐raising allele 1.05; 95% CI: 1.00, 1.10). Using rs11977526 as an IV we estimated the causal effect of a one SD increase in IGF‐II (∼265 ng/mL) on risk of high *vs*. low grade disease as 1.14 (95% CI: 1.00, 1.31). Because of the potential for pleiotropy of the genetic instruments, these findings can only causally implicate the IGF pathway in general, not any one specific biomarker.

## Abstract 2

**What's New?**

Circulating insulin‐like growth factors (IGF) and their binding proteins have been associated with prostate cancer risk in observational epidemiological studies but it is not clear whether there is a causal relationship with disease. To address this question, the authors used Mendelian randomization, a method that uses genetic variants as proxies for measured exposures. Their results implicate the IGF pathway in general in prostate cancer development but specific biomarkers remain to be determined.

**Abbreviations**
BPH: benign prostatic hyperplasia
CV: coefficient of variation
GWAS: genome‐wide association study
IBD: identity by descent
ICC: intra‐class correlation
IGF: insulin‐like growth factor
IGFBP: insulin‐like growth factor‐binding proteins
IV: instrumental variable
LD: Linkage disequilibrium
MR: Mendelian randomization
PSA: prostate specific antigen
QC: quality control
RIA: radioimmunoassay
SD: standard deviation
SNP: single nucleotide polymorphism
UKHLS: *UK Household Longitudinal Study*.

Prostate cancer is the most common male cancer in industrialised countries, yet there are no established, potentially modifiable risk factors for prevention.^1^ The nutritionally regulated IGFs, and their modulating binding proteins (IGFBPs) play a key role in somatic growth, and activate carcinogenic intracellular signalling networks.^1^ Meta‐analyses of epidemiological studies generally observe positive associations of circulating IGF‐I with prostate cancer,^2^, ^3^, ^4^ but substantial differences exist between studies.^5^, ^6^

Such diverse evidence indicates that causation remains to be established. Alternative explanations for the observed association of IGF‐axis peptides with prostate cancer include: reverse causality, because tumours may promote an endocrine response^7^; confounding by dietary,^8^ nutritional^9^ and lifestyle^10^ factors; measurement error,^11^ as single serum measurements may inadequately reflect long‐term exposure; or detection bias,^11^ occurring, for example, if IGF‐I causes symptomatic benign prostatic hyperplasia (BPH) that results in the serendipitous finding of latent cancer on diagnostic biopsy.

Mendelian randomization (MR)^12^ seeks to establish causality by using genetic variants as proxies for the exposure of interest. Since alleles randomly assort at gamete formation and segregate randomly at conception to generate genotypes, associations between genotypes and outcome are not generally confounded by behavioural or environmental factors and cannot be explained by reverse causation. Genetic variation may also be a better measure of exposure over a lifetime than a single serum measurement, as those with genotypes causing high (or low) IGF levels will have been, in effect, randomly allocated to high (or low) IGF levels from birth. To determine causality, MR relies on an association between genetic variant (also known as instrument) and exposure so that the greater the correlation between the two, and thus the more variation in the exposure phenotype explained by the genotype, the more reliable the causal inference. Additionally, the instrument is expected to influence the outcome only via the exposure (*i.e*., absence of horizontal pleiotropy^13^) and to be independent from confounders of the relationship between exposure and outcome.

We used an MR approach in an attempt to assess the causal role of the IGF axis in prostate cancer. First, we validated genetic variants previously associated with IGF levels in the literature to confirm reported associations of the SNPs (especially SNPs selected from candidate gene studies), and to assess the potential for pleiotropic effects of the genetic variants on more than one IGF protein. Second, we performed a large case–control study based on an international prostate cancer consortium of >22,000 case/control pairs using the validated polymorphisms.

## Material and Methods

### Study populations

#### ProtecT (Prostate testing for cancer and Treatment) study

The association of genetic variants with IGF levels was evaluated in the control arm of a case–control study nested within ProtecT, a UK multicentre study to identify localised prostate cancer and evaluate its management in a randomly allocated controlled trial.^5^ All men without evidence of prostate cancer were eligible for selection as controls; that is, men with a prostate specific antigen (PSA) test < 3 ng/mL, or men with a raised PSA (≥ 3 ng/mL) combined with at least one negative diagnostic biopsy. Of the 2,766 controls who underwent measures of IGFs in ProtecT^5^, ∼700 men also had genome‐wide genotype data available (mean age ± SD: 62.1 ± 5.0 years).

Blood samples for IGF measurement in ProtecT were drawn at the time of the PSA test, frozen at −80°C within 36 hr, then transferred on dry ice for assay.^4^ Total IGF‐I, IGF‐II and IGFBP‐3 levels were measured by in‐house radioimmunoassay (RIA) and circulating IGFBP‐2 was measured using a one‐step sandwich ELISA (DSL‐10–7100; Diagnostic Systems Laboratories). The intra‐class correlations (ICC) for within‐assay variability for IGF‐I, IGF‐II, IGFBP‐2 and IGFBP‐3 were 0.86, 0.91, 0.95 and 0.88; the ICCs for between‐assay variability were 0.66, 0.84, 0.81 and 0.71, respectively.

Genome‐wide genotyping of participants was carried out at the Centre National de Génotypage (CNG, Evry, France), using the Illumina Human660W‐Quad_v1_A array (Illumina Inc., San Diego, CA). The quality control (QC) process performed before imputation excluded individuals on the basis of the following: sex mismatches, minimal (< 0.325) or excessive (> 0.345) heterozygosity, disproportionate levels of individual missingness (> 3%), cryptic relatedness measured as a proportion of identity by descent (IBD > 0.1), and insufficient sample replication (IBD < 0.8). All individuals with non‐European ancestry, and SNPs with a minor allele frequency (MAF) below 1%, a call rate of < 95% or out of Hardy‐Weinberg equilibrium (*p* < 5 × 10^−7^) were removed. Autosomal genotypic data were imputed using Markov Chain Haplotyping software (MACH v.1.0.16)^14^ and phased haplotype data from European (CEU) individuals (HapMap release 22, Phase II NCBI B36, dbSNP 126) based on 514,432 autosomal SNPs. After imputation, all SNPs with indication of poor imputation quality (*r* ^2^ hat < 0.3) were eliminated. The working dataset consisted of 2,927 individuals (1,136 cases, 1,791 controls) of European ancestry.

Trent Multicenter Research Ethics Committee (MREC) approved both the ProtecT study (MREC/01/4/025), and the associated ProMPT study which collected biological material (MREC/01/4/061). Written informed consent was obtained from all men.

#### ALSPAC (Avon Longitudinal Study of Parents and Children)

We used ALSPAC to replicate ProtecT findings. ALSPAC is a population‐based prospective cohort study of children and their parents. The study is described in detail elsewhere^15^, ^16^, ^17^ (http://www.bristol.ac.uk/alspac/). Measurement of circulating IGF‐I, IGF‐II and IGFBP‐3 in plasma or serum was carried out as in ProtecT. IGFBP‐2 was not measured. The intra‐ and inter‐assay coefficients of variation (CV) were 7.0 and 14.3% for IGF‐ I, 7.9 and 18.6% for IGF‐II, and 6.1 and 8.7% for IGFBP‐3.^18^

Genome‐wide association study (GWAS) data for the children were generated by Sample Logistics and Genotyping Facilities at the Wellcome Trust Sanger Institute (Cambridge, UK) and the Laboratory Corporation of America (Burlington, NC, USA) with support from 23andMe (Mountain View, CA, USA) using the Illumina HumanHap550 quad chip. The mothers were genotyped at CNG using the Illumina Human660W quad array. All individuals of non‐European ancestry, ambiguous sex, extreme heterozygosity, cryptic relatedness (IBD > 0.1 in children, > 0.125 in mothers), high missingness (> 3% in children, > 5% in mothers) and insufficient sample replication (IBD < 0.8) were removed. SNPs with genotyping rate < 95%, MAF < 1%, or out of Hardy‐Weinberg equilibrium (*p* < 5 × 10^−7^ in children, *p* < 1 × 10^−6^ in mothers) were excluded. Genotypic data was subsequently phased with ShapeIT v2.r644,^19^ and imputed using IMPUTE v2.2.2^20^ and phased haplotype data from the 1000 Genomes reference panel (phase 1, version 3), based on 465,740 SNPs. The cleaned dataset consisted of 8,237 children and 8,196 mothers. Up to ∼400 pregnant women (mean ± SD age at delivery: 28.7 ± 5.4 years) and ∼450 children at different ages (mean ± SD age: 61.8 ± 0.8 months, 54% male; 7.5 ± 0.2 years, 54% male; 8.2 ± 0.1 years, 56% male), as well as ∼500 umbilical cord samples had genotypes and IGF measures for analysis.

Ethical approval for the study was obtained from the ALSPAC Ethics and Law Committee and the Local Research Ethics Committees (http://www.bristol.ac.uk/alspac/researchers/data-access/ethics/lrec-approvals/#d.en.164120). Written informed consent was obtained from all participants in the study.

#### Understanding Society: the UK Household Longitudinal Study (UKHLS)

SNPs validated in ProtecT were also examined in the UKHLS study, which is a stratified clustered random sample of households, representative of the UK population (https://www.understandingsociety.ac.uk/). Serum IGF‐I levels were measured using an electrochemiluminescent immunoassay on an IDS ISYS analyser. The inter‐ and intra‐assay C *vs*. were < 14%. No measurements of IGF‐II, IGFBP‐2 or IGFBP‐3 were available.

In total, 10,480 samples were genotyped on the Illumina HumanCoreExome chip (v1.0) at the Wellcome Trust Sanger Institute. Data QC was performed at the sample‐level using the following filters: call rate < 98%, autosomal heterozygosity outliers (> 3 SD), gender mismatches, duplicates as established by IBD analysis (PI_HAT > 0.9), ethnic outliers. Variants with a Hardy‐Weinberg equilibrium *p* values < 10^−4^, a call rate below 98% and poor genotype clustering values (< 0.4) were removed, as well as mitochondrial polymorphisms, leaving 518,542 variants. Imputation was performed at the UCL Genetics Institute using Minimac version 5–29‐12,^21^ MaCH^14^ for phasing, and the 1000 Genomes Project, March 2012, version 3, NCBI build GRCh37/hg19 as a reference sample. The final sample consisted of 9,944 individuals. As UKHLS is a household study we additionally eliminated individuals who were related (> 5%), thus the working sample included 9,237 participants (mean ± SD age: 54.1 ± 16.1 years, 44% male).

UKHLS is designed and conducted in accordance with the ESRC Research Ethics Framework and the ISER Code of Ethics. The University of Essex Ethics Committee approved waves 1–5 of UKHLS. Approval from the National Research Ethics Service was obtained for the collection of biosocial data by trained nurses in waves 2 and 3 of the main survey (Oxfordshire A REC, Reference: 10/H0604/2).

#### PRACTICAL Consortium (PRostate cancer AssoCiation group to Investigate Cancer‐Associated aLterations in the genome)

We investigated associations of published IGF‐related genetic variants, evaluated as instruments in ProtecT and replicated in ALSPAC and/or UKHLS, with prostate cancer risk, progression and mortality in men from 25 studies contributing to the international PRACTICAL consortium^22^ (http://practical.ccge.medschl.cam.ac.uk). Seventeen studies were from Europe, six from North America and two from Australia, and comprised population samples of predominantly European ancestry^22^ (Table 1). Data on cancer stage, grade and method of diagnosis were collected by each study using a variety of methods. We categorised cancers as localised (T1 or T2 on TNM staging, or if not available, “localised” on SEER staging) or advanced (T3 or T4, or “regional” or “distant” on SEER staging).

**Table 1:** Clinical characteristics of prostate cancer cases in 25 PRACTICAL studies

Study	Country	*N* controls	*N* cases	Mean age at diagnosis (years)	Mean PSA at diagnosis (ng/mL)	European ethnicity (%)^a^	Family history of prostate cancer (%)^1^, ^2^	High Gleason score (≥7, %)^a^	Advanced stage (%)^1^, ^3^	Screen‐detected cancer (%)^a^
**CAPS**	Sweden	664	1153	66.1	79.6	100	17.4	49.9^b^	30.3	0.0
**CPCS1**	Denmark	2756	848	69.5	48.0	99.6	8.2^b^	71.2^b^	n/a	0.0
**CPCS2**	Denmark	1001	265	64.9	36.0	99.4	14.7^b^	52.2^b^	n/a	0.0
**EPIC**	Europe	1079	722	64.9	0.2	100	n/a	27.9^b^	4.0^b^	0.0
**EPIC‐Norfolk**	UK	911	481	72.1	n/a	99.9	2.5	39.4^b^	n/a	n/a
**ESTHER**	Germany	318	313	65.5	58.7	100	8.9^b^	48.0	27.6	61.9^b^
**FHCRC**	USA	729	761	59.7	16.1	99.9	21.7	41.7	20.2	N/a
**IPO‐Porto**	Portugal	66	183	59.3	8.3	100	20.0^b^	84.2	64.5	82.8^b^
**MAYO**	USA	488	767	65.2	15.5	100	29.1	55.3^b^	45.5	73.7^b^
**MCCS** c	Australia	1169	1650	58.5	18.8	98.8	23.5^b^	53.4	14.5	N/a
**MEC**	USA	829	819	69.5	n/a	100	13.0	n/a	12.5	N/a
**MOFFITT**	USA	96	404	65.0	7.3	97.5	22.3	43.4	3.6	0.0^b^
**PCMUS**	Bulgaria	140	151	69.3	32.5	100	5.3	59.6	46.7	21.2
**Poland**	Poland	359	438	67.7	40.2	100	10.6	32.8^b^	37.1^b^	0.0^b^
**PPF‐UNIS**	UK	187	244	68.9	32.1	99.8	25.3	45.2^b^	28.8^b^	N/a
**ProMPT**	UK	2	166	66.3	33.0	100	34.6	74.3^b^	34.7	0.0^b^
**ProtecT**	UK	1458	1545	62.7	9.6	99.7	8.0^b^	29.9	11.4	100.0
**QLD**	Australia	85	139	61.4	7.4	99.1^d^	37.8	83.6	0.0^b^	N/a
**SEARCH**	UK	1231	1354	63.1	53.2	100	16.3	56.9^b^	18.0^b^	36.7^b^
**STHM1**	Sweden	2224	2006	66.2	n/a	100	20.2	45.5^b^	14.4^b^	N/a
**TAMPERE**	Finland	2413	2754	68.2	69.2	100	n/a	43.8^b^	21.4	46.8
**UKGPCS**	UK	4132	3838	63.6	88.0	99.8	22.4^b^	50.5^b^	36.4^b^	28.0^b^
**ULM**	Germany	354	603	63.8	19.1	100	44.9	51.3^b^	40.5	N/a
**UTAH**	USA	245	440	62.6	n/a	100	51.4	n/a	17.2^b^	N/a
**WUGS**	USA	0	948	60.8	6.1	95.8	42.6^b^	59.3	24.2	N/a

*N* = 45,928 men.

Information in the table is given for the subset of individuals whose ethnicity was “European” (except for the study's European ethnicity percentage).

a Percent of cases with data available.

Family history of prostate cancer in a first degree relative.

T3 or T4 on TNM staging, or if not available, “regional” or “distant” on SEER staging.

b Information missing for >10% of patients.

c MCCS includes Risk Factors for Prostate Cancer Study (RFPCS) and The Early Onset Prostate Cancer Study (EOPCS).

d Information missing for >10% of individuals.

n/a not available.

Genotyping of PRACTICAL samples was carried out using an Illumina Custom Infinium genotyping array (iCOGS), designed for the Collaborative Oncological Gene‐Environment Study (COGS) (http://www.cogseu.org /) and consisting of 211,155 SNPs.^22^ This array was devised to evaluate associations of genetic variants with breast, ovarian and prostate cancer (85,278 were specifically chosen for their potential relevance to prostate cancer). A total of 201,598 SNPs passed QC for the European ancestry samples.^22^ Imputation of ∼17 million SNPs/indels using the 1000 Genomes Project (version 3, March 2012 release) as a reference panel was performed with the program IMPUTE v.2.^20^ Polymorphisms with quality information scores of (*r* ^2^) > 0.3 and MAF > 0.5 were taken forward for analysis.^23^ Overall there were 22,992 prostate cancer cases and 22,936 controls with genotype data available.

All studies have the relevant Institutional Review Board approval in each country in accordance with the Declaration of Helsinki.

### Identification of genetic variants associated with IGF levels in the literature

We selected single nucleotide polymorphisms (SNPs) associated with circulating IGF levels from the National Human Genome Research Institute‐European Bioinformatics Institute (NHGRI‐EBI) catalog of genome‐wide association studies (GWAS) (https://www.ebi.ac.uk/gwas/) and by conducting a PubMed literature search. All SNPs chosen were associated with IGF concentration at the significance thresholds established by each study (*p* < 5 × 10^**−**^^7^ in the discovery GWAS; usually *p* < 0.05 in candidate gene studies).

### Validation of genetic variants as instruments of IGF levels

The properties of the SNPs as instrumental variables (IV) were assessed in ProtecT controls by examination of: (i) *F* statistics (with values lower than 10 taken as evidence of a weak instrument^24^) and *R^2^* values (the proportion of variation in IGF levels explained by the genetic variant) from the linear regression of each biomarker on the SNP; (ii) associations of the genetic variants with potential confounding factors and other variables (age, PSA at recruitment, body mass index (BMI), height, leg‐length, BPH and diabetes); and (iii) possible pleiotropic effects of the variants on more than one IGF peptide.^25^ The validated genetic instruments were tested for replication in ALSPAC mothers and children, and UKHLS participants.

### Statistical analysis

All SNPs were examined for deviation from Hardy‐Weinberg equilibrium using the hwsnp function in the statistical package Stata. Linear and logistic regression were used as appropriate to investigate the effect of SNPs on IGF‐I, IGF‐II, IGFBP‐2, IGFBP‐3, PSA and potential confounders. For the validated SNPs we ran meta‐analyses across all PRACTICAL studies to evaluate between‐study heterogeneity in the association with prostate cancer risk, Gleason grade (low: <7 *vs*. high: ≥ 7) and stage (localised *vs*. advanced). We computed pooled ORs assuming a fixed‐effects model when there was no evidence of heterogeneity (*p* > 0.05), otherwise we used a random‐effects model. Logistic regression with robust standard errors, to account for within‐study clustering, was performed to test for associations of all polymorphisms across the *IGFBP1/IGFBP3* region and SNPs in other chromosomal regions with the above prostate cancer outcomes.

Linkage disequilibrium (LD) between pairs of variants in the *IGFBP‐1/IGFBP‐3* gene region was calculated with the program LDlink using data for the GBR population (English and Scottish) in Phase 3 of the 1,000 Genomes Project.^26^ *r* ^2^ values obtained with LDlink were then used to create an LD plot of the region with the R package LDheatmap (http://www.R-project.org). Functional consequences of genetic polymorphisms were predicted using SNPnexus (http://www.snp-nexus.org/).

#### Survival analysis

Amongst men with prostate cancer, we estimated associations of the validated SNPs with long‐term (15‐year) survival, examining all‐cause and prostate cancer‐specific mortality using Cox proportional hazards regression with date at diagnosis as the start date and date at death or final follow‐up time‐point as the exit date, with robust standard errors to account for within‐study clustering.

#### Instrumental variable (IV) analysis

**Figure 1:**
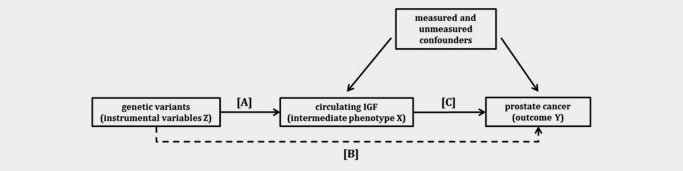
Directed acyclic graph (DAG) showing the instrumental variable (IV) assumptions underpinning a Mendelian randomization analysis of circulating IGF levels with prostate cancer. IV models use associations A and B to estimate the causal effect of IGF on prostate cancer C (C = B/A). The instrument is assumed not to have a direct effect on the outcome, hence the dashed line is to illustrate that association B is required for IV estimation. The effect of genotype on the outcome should be mediated only through the intermediate phenotype (no pleiotropy). The numerator of the two sample IV estimator is the log odds ratio from a logistic regression of the outcome (Y) on the instrument (Z) in the PRACTICAL population and the denominator is the beta coefficient from a linear regression of the exposure (X) on the instrument (Z) in the ProtecT or UKHLS population or obtained from the literature.

To estimate the causal effect of IGF levels on prostate cancer, we used validated SNPs as the instruments in a two‐sample ratio estimator IV analysis^27^, ^28^ (Fig. 1). The ratio represents the causal log odds ratio of a one unit increase in circulating IGF on the risk of prostate cancer. IV analysis was conducted for the SNPs showing the strongest association with prostate cancer, which were also associated with circulating IGFs in ProtecT, ALSPAC or UKHLS, and the estimates are given per standard deviation (SD) increase in IGF levels.

#### Adjustments

Principal components reflecting each population's genetic structure were included as covariates in the regression models to account for confounding by population stratification. Additional adjustments for age at diagnosis, age at blood sample collection, gestational age and sex were made when appropriate.

Unless otherwise specified, all analyses were carried out in Stata version 13 (StataCorp LP, 2013, College Station, TX).

## Results

We identified 56 SNPs that were associated with circulating IGF peptides in GWAS (*n* = 8) or candidate gene studies (*n* = 48) (Supporting Information Table 1). Most of these SNPs were located in the *IGF1* and *IGFBP1*/*IGFBP3* gene regions on chromosomes 12q23.2 and 7p12.3, respectively, and showed associations with IGF‐I and IGFBP‐3 levels. We could only find one candidate gene study that had examined the relationship of blood IGF‐II with genetic polymorphisms,^29^ and one that had similarly considered IGFBP‐2 concentrations.^30^

### Validation of the association of published SNPs with IGF levels in ProtecT controls

IGF‐I, IGF‐II and IGFBP‐3 blood concentrations were approximately normally distributed, as opposed to IGFBP‐2, which was natural log‐transformed for analysis. Mean (± SD) levels are given in Supporting Information Table 2. All SNPs, with the exception of rs3770473 (*p* < 0.0001), conformed with Hardy‐Weinberg equilibrium. Six SNPs in the *IGFBP1/IGFBP3* gene region were strongly associated with circulating IGFs (F‐statistic > 10),^31^ individually explaining ∼2 – 5% of variation in biomarker concentration (Table 2). The genetic variant showing the strongest association, and thus ranking as the best instrument, was rs11977526 (F = 38, R^2^ = 5%), the lead SNP in a GWAS of IGF‐I and IGFBP‐3 levels.^32^ Five out of the six SNPs (including rs11977526) were not associated with the IGF biomarker reported in the literature but with IGF‐II instead. Only one SNP (rs700752) was consistent with published reports, showing associations with both IGF‐I and IGFBP‐3 (although it qualified as a strong instrument only for IGFBP‐3) (Table 2). Three of the most robustly associated variants (rs11977526, rs1496499, rs700752) had been identified in a GWAS including over 10,000 participants,^32^ and the remaining three (rs3110697, rs2132571, rs924140) were in strong LD with the first two (Supporting Information Fig. 1).

**Table 2:** Association of published SNPs with IGF biomarkers in ProtecT controls

			ProtecT: effect on published biomarkers			ProtecT: effect on other biomarkers					
SNP	Effect allele/non‐effect allele^a^	Published associations	Mean difference in IGF levels (ng/mL) per effect allele	95% CI	*p* value	Other associations	Mean difference in IGF levels (ng/mL) per effect allele	95% CI	*p* value	F	R^b^ (%)
rs3770473	G/T	IGF‐I	1.06	(−8.77,10.89)	0.83						
		IGFBP‐3	−43.89	(−225.24,137.47)	0.64						
rs300982	G/A	IGFBP‐3	−139.80	(−420.66, 141.05)	0.33						
**rs4234798**	T/G	IGFBP‐3	−49.51	(−165.48,66.45)	0.40						
rs7703713	A/G	IGF‐I	−1.32	(−8.14,5.49)	0.70	IGFBP‐2	−0.07	(−0.14, −0.001)	0.04	2.5	0.34
**rs2153960**	A/G	IGF‐I	3.67	(−3.16,10.49)	0.29	IGFBP‐2	0.07	(0.002,0.14)	0.04	3.6	0.50
rs998075	G/A	IGF‐I	1.78	(−4.14,7.71)	0.56						
rs998074	C/T	IGF‐I	1.78	(−4.14,7.71)	0.56						
**rs7780564**	C/A	IGF‐I	4.35	(−1.46,10.15)	0.14						
rs10228265	A/G	IGFBP‐3	−11.25	(−126.51,104.00)	0.85	IGF‐II	27.31	(−1.71,56.33)	0.07	3.8	0.52
rs1908751	T/C	IGF‐I	−0.40	(−6.98,6.18)	0.91						
rs2270628	C/T	IGFBP‐3	3.35	(−129.87,136.56)	0.96	IGF‐II	34.97	(1.40,68.54)	0.04	4.9	0.68
rs6670	T/A	IGF‐I	−5.62	(−14.58,3.35)	0.22						
rs3110697	G/A	IGFBP‐3	−34.10	(−144.90,76.69)	0.55	IGF‐II	55.26	(27.60,82.92)	9.64x10^−5^	14.3	1.94
rs9282734	G/T	IGFBP‐3	360.75	(−574.69,1296.20)	0.45						
rs2471551	G/C	IGFBP‐3	7.96	(−128.43,144.34)	0.91	IGF‐I	9.03	(−1.65,16.42)	0.02	5.6	0.76
						IGF‐II	−44.24	(−78.55, −9.93)	0.01	6.0	0.82
rs2132572	C/T	IGFBP‐3	−52.69	(−180.87,75.48)	0.42	IGF‐II	35.09	(2.79,67.38)	0.03	4.3	0.59
		IGF‐I	−4.32	(−11.30,2.65)	0.22						
rs2132571	C/T	IGFBP‐3	62.68	(−53.82,179.19)	0.29	IGF‐II	55.35	(26.15,84.55)	2.14x10^−4^	11.6	1.58
						IGF‐I	6.79	(0.45,13.13)	0.04	4.0	0.54
rs924140	T/C	IGFBP‐3	13.33	(−97.43,124.10)	0.81	IGF‐II	76.49	(49.08,103.89)	5.92x10^−8^	26.0	3.47
**rs1496499**	G/T	IGF‐*I* b	3.12	(−2.48,8.72)	0.27	IGF‐II	77.18	(49.81,104.55)	4.35x10^−8^	26.3	3.52
**rs11977526**	A/G	IGFBP‐3	83.98	(−31.18,199.14)	0.15	IGF‐II	94.78	(66.48,123.09)	9.53x10^−11^	37.8	4.98
		IGF‐I^b^	3.07	(−2.77,8.91)	0.30						
**rs700752**	G/C	IGF‐I	9.22	(3.19,15.24)	0.003					7.7	1.05
		IGFBP‐3	219.21	(108.61,329.81)	1.09x10^−4^					13.6	1.86
**rs1245541**	G/A	IGF‐I	−0.79	(−6.93,5.34)	0.80						
rs217727	A/G	IGF2	14.65	(−20.09,49.39)	0.41	IGFBP‐3	135.16	(−2.00,272.33)	0.053	2.0	0.28
rs6214	T/C	IGF‐I	2.64	(−3.51,8.79)	0.40						
rs1520220	G/C	IGF‐I	6.37	(−1.88,14.61)	0.13						
rs5742694	A/C	IGF‐I	−5.59	(−12.74,1.56)	0.13						
rs978458	T/C	IGF‐I	5.22	(−1.79,12.23)	0.14						
rs5742678	C/G	IGF‐I	5.22	(−1.79,12.23)	0.14						
rs972936	C/T	IGF‐I	−5.22	(−12.23,1.79)	0.14						
rs2288378	T/C	IGF‐I	5.60	(−1.55,12.74)	0.12						
rs7136446	C/T	IGF‐I	3.81	(−2.19,9.81)	0.21						
rs10735380	G/A	IGF‐I	6.13	(−0.71,12.96)	0.08					3.4	0.47
rs2195239	G/C	IGF‐I	5.89	(−1.35,13.13)	0.11						
rs12821878	G/A	IGF‐I	6.93	(−0.18,14.05)	0.06					3.2	0.43
rs5742615	T/G	IGF‐I	3.99	(−28.62,36.60)	0.81						
rs2162679	T/C	IGFBP‐3	−38.78	(−201.52,123.96)	0.64						
rs5742612	G/A	IGF‐I	−8.36	(−25.99,9.26)	0.35						
		IGFBP‐3	−81.73	(−409.99,246.53)	0.63						
rs35767	A/G	IGF‐I	1.27	(−7.47,10.01)	0.78						
		IGFBP‐3	38.78	(−123.96,201.52)	0.64						
rs35766	C/T	IGF‐I	3.58	(−4.85,12.02)	0.41						
rs35765	T/G	IGF‐I	6.45	(−3.14,16.04)	0.19						
rs7965399	C/T	IGF‐I	−4.86	(−20.59,10.86)	0.54						
rs11111285	G/A	IGF‐I	−4.96	(−20.73,10.80)	0.54						
		IGFBP‐2	0.003	(−0.15,0.16)	0.97						
rs855211	A/G	IGF‐I	2.50	(−5.75,10.75)	0.55						
rs10778177	C/T	IGF‐I	−1.22	(−9.80,7.36)	0.78						
rs855203	C/A	IGF‐I	2.52	(−7.95,12.99)	0.64						
rs1457596	A/G	IGF‐I	2.94	(−7.83,13.71)	0.59						
rs7964748	A/G	IGF‐I	1.25	(−6.44,8.94)	0.75						
rs907806	G/A	IGFBP‐3	−112.72	(−285.09,59.66)	0.20						
rs213656	T/G	IGF‐I	4.32	(−1.66,10.30)	0.16	IGFBP‐2	−0.06	(−0.12,0.00)	0.05	4.1	0.56
rs3751830	C/T	IGF‐I	3.23	(−2.80,9.26)	0.29	IGFBP‐2	−0.05	(−0.11,0.01)	0.09	3.3	0.44
rs197056	A/G	IGF‐I	6.70	(0.61,12.78)	0.03	IGFBP‐2	−0.06	(−0.12,0.00)	0.06	3.6	0.50
rs174643	G/A	IGF‐I	4.24	(−1.64,10.11)	0.16	IGFBP‐2	−0.05	(−0.11,0.01)	0.09	3.3	0.45
rs1178436	C/T	IGFBP‐3	188.47	(45.27,331.67)	0.01					7.1	0.98
**rs1065656**	G/C	IGFBP‐3	146.47	(27.31,265.63)	0.02					5.3	0.73
rs17559	A/G	IGFBP‐3	100.90	(−74.95,276.75)	0.26						
rs11865665	G/A	IGFBP‐3	164.35	(−40.99,369.68)	0.12						

a The effect allele is expected to increase the levels of biomarkers reported in the literature.

b IGF‐I adjusted for IGFBP‐3.

Circulating IGFBP‐2 was natural log transformed.

The regression models were adjusted for age and 10 principal components.

IGF‐I *N* = 727, IGF‐II *N* = 718, IGFBP‐2 *N* = 724, IGFBP‐3 *N* = 712.

In bold, SNPs uncovered in a GWAS of IGF‐I and IGFBP‐3 levels.

Other SNPs identified in the same GWAS, but located in different chromosomal regions, were either not associated with the serum concentration of any biomarker (rs4234798, rs7780564 and rs1245541), marginally associated with a biomarker other than the one reported in the GWAS (rs2153960 with IGFBP‐2 instead of IGF‐I), or showed an association with the GWAS‐reported biomarker (IGFBP‐3) but did not satisfy the requirements of a strong instrument (rs1065656) (Table 2).

The validated SNPs were not correlated with potential confounders or PSA, after applying a Bonferroni correction for multiple testing (*p* values > 0.001) (Supporting Information Table 3).

### Replication in ALSPAC

Mean (± SD) levels of IGF‐I, IGF‐II and IGFBP‐3 for mothers and children are shown in Supporting Information Table 2. All SNPs that were strong instruments for IGF‐II in ProtecT (rs11977526, rs1496499, rs2132571, rs3110697, rs924140) plus two extensively studied functional variants rs2854744 (−202 A/C) and rs2854746 (Gly32Ala) that were not genotyped or imputed in ProtecT and are in strong LD with rs11977526 (r^2^ = 0.66 for rs2854744 and 0.98 for rs2854746 in the UK population), were replicated with respect to IGF‐II levels in ALSPAC. The strongest instruments were: rs2854746, explaining between 2.5% (in cord blood samples) and 11.4% (in 61 month‐old children) of variation in IGF‐II; and rs11977526, explaining 4.3% of variation in maternal IGF‐II. Unlike in ProtecT, and in agreement with the literature, these SNPs were generally also associated with IGFBP‐3 levels, although not as strongly as with IGF‐II. The strongest instruments for IGFBP‐3 were rs2854746 (R^2^ = 4.9% in mothers), rs1496499 (R^2^ = 6.1% in children) and rs700752 (R^2^ = 4.1% in children) (Supporting Information Table 4). No strong associations with IGF‐I were uncovered. SNPs identified in the discovery GWAS, not on 7p12.3, were weakly or not at all (rs7780564) associated with IGF levels (Supporting Information Table 5).

### Replication in UKHLS

Mean (± SD) IGF‐I concentrations for men and women who participated in UKHLS are shown in Supporting Information Table 2, whilst association results are displayed in Supporting Information Table 4. All SNPs, with the exception of rs2132571, were associated with serum IGF‐I. SNPs that were in strong LD (*i.e.,* all excluding rs700752) showed associations consistent with those reported in the literature, although in the literature their effects were adjusted for IGFBP‐3 levels, which we could not do in UKHLS as circulating IGFBP‐3 was not available. Variants rs700752, rs11977526 and rs2854746 qualified as strong instruments for IGF‐I levels (*F* > 10) but did not appear to explain much of the variance in the trait. Results for other GWAS‐identified variants can be found in Supporting Information Table 5.

### Association of validated SNPs with prostate cancer risk and progression in PRACTICAL

Fixed‐effects and random‐effects meta‐analyses of the eight validated polymorphisms identified stronger associations with prostate cancer grade than with risk or disease stage (Table 3). Rs11977526 (the strongest instrument) was associated with high Gleason grade (OR per A allele 1.05; 95% CI: 1.00, 1.10) (Supporting Information Fig. 2). This variant's A (minor) allele was associated with increased IGF‐II levels in ProtecT and ALSPAC, IGFBP‐3 levels in the literature and ALSPAC, and with reduced IGF‐I levels in UKHLS. Other SNPs in the region in LD with rs11977526 had a similar effect on disease grade (Table 3). The major allele in rs700752, which is associated with higher IGF‐I levels, showed a weakly protective effect with respect to high grade prostate cancer (OR per G allele 0.97; 95% CI: 0.92, 1.01) (Supporting Information Fig. 3). Evidence of association is limited when a Bonferroni correction for multiple testing is applied.

**Table 3:** SNPs associated with IGF levels in ProtecT and prostate cancer risk, grade and stage in the PRACTICAL consortium

SNP	Chromosome position^a^	Effect/non‐effect allele^b^	OR case–control^c^	95% CI	*p* value	*I* b (%)	OR Gleason grade^d^	95% CI	*p* value	*I* b (%)	OR stage^e^	95% CI	*p* value	*I* b (%)
rs3110697	7:45915430	G/A	1.00	(0.97,1.04)	0.79	0.0	1.06	(0.98,1.14)	0.15^f^	55.3	1.03	(0.98,1.08)	0.26	32.4
rs2854746	7:45921046	C/G	1.02	(0.99,1.05)	0.28	0.0	1.04	(0.99,1.08)	0.13	6.0	1.00	(0.93,1.08)	0.93^f^	39.7
rs2854744	7:45921476	A/C	1.01	(0.98,1.05)	0.39	17.4	1.04	(1.00,1.09)	0.08	20.6	1.02	(0.96,1.07)	0.56	30.6
rs2132571	7:45922075	C/T	1.00	(0.97,1.04)	0.88	28.7	1.03	(0.98,1.08)	0.26	0.0	1.00	(0.94,1.05)	0.85	0.0
rs924140	7:45923515	T/C	1.01	(0.98,1.05)	0.37	30.6	1.04	(1.00,1.09)	0.08	19.0	1.02	(0.95,1.10)	0.63^f^	37.0
rs1496499	7:45939424	G/T	1.01	(0.96,1.06)	0.69^f^	41.7	1.05	(1.01,1.10)	0.03	17.5	1.03	(0.98,1.08)	0.32	33.4
rs11977526	7:45968511	A/G	1.01	(0.98,1.05)	0.41	16.3	1.05	(1.00,1.10)	0.06	11.8	1.04	(0.98,1.10)	0.17	33.3
rs700752	7:46713955	G/C	1.00	(0.97,1.04)	0.85	0.0	0.97	(0.92,1.01)	0.17	0.0	0.97	(0.92,1.03)	0.30	0.0

Fixed‐effects and random‐effects meta‐analyses adjusted for age and 15 principal components.

a GRCh38.p2.

b The effect allele is expected to increase the levels of biomarkers reported in the literature.

c 22 studies included.

d Gleason grade: <7 *vs*. ≥7. 23 studies included.

e Stage: localised *vs*. advanced. 21 studies included.

f Random effects meta‐analysis.

19,071 cases/19,994 controls.

9,429 low grade (<7)/8,913 high grade (≥7) disease.

14,201 localised/4,455 advanced disease.

### Survival analysis in PRACTICAL

Rs700752 was associated with prostate cancer‐specific mortality, with the allele that increases IGF‐I and IGFBP‐3 levels (major) being associated with a lower risk of death. No other associations with all‐cause or prostate cancer‐specific mortality were observed, except when considering the non‐additive relationship of the genetic variant with survival (Supporting Information Table 6). In the case of SNPs linked to rs11977526 (*i.e.,* rs1496499, rs2854744, rs2854746 and rs924140) heterozygotes exhibited the highest mortality rates, compared to homozygotes. The proportional hazards assumption was not fulfilled for many of the variants (*p* < 0.05).

### Instrumental variable analysis

An IV analysis using individual‐level data was run for rs11977526 and IGF‐II, as it had been genotyped/imputed in both ProtecT and PRACTICAL, and showed associations with circulating IGF‐II in ProtecT and prostate cancer grade in PRACTICAL. The estimated causal OR per one SD (∼265 ng/mL) increase in serum IGF‐II was 1.14 (95% CI: 1.00, 1.31) for high (*vs*. low) grade disease. Similarly, using information from UKHLS on the association between rs11977526 and IGF‐I, we estimated a causal OR of 0.39 (95% CI: 0.14, 1.10) per one SD (∼50 ng/mL) increase in circulating IGF‐I for high Gleason grade cancer.

We used summary data for the association of rs11977526 with IGFBP‐3 from the discovery GWAS^32^ (results from the Framingham Heart Study cohort as the largest study) and its association with Gleason grade in PRACTICAL, to estimate the causal OR per one SD (∼1000 ng/mL) increase in IGFBP‐3 as 1.15 (95% CI: 1.00, 1.32) for high (*vs*. low) grade disease.

Finally, if rs700752 is employed as an IV for serum IGF‐I and IGFBP‐3, based on ProtecT findings, the causal estimates regarding prostate cancer‐specific mortality were HR 0.72 (95% CI: 0.53, 0.98) per SD increase in IGF‐I, and HR 0.76 (95% CI: 0.60, 0.95) per SD increase in IGFBP‐3. Considering UKHLS as the source of the SNP‐exposure effect, the causal estimate per SD increase in IGF‐I levels was lower but comparable, HR 0.47 (95% CI: 0.29, 0.82).

### Further analysis (see Supporting Information Results)

In order to obtain a more complete picture of the *IGFBP1/IGFBP3* genetic region and its relationship to prostate cancer, we carried out an analysis of all additional SNPs within these genes that were available in PRACTICAL (*n* = 39).

We also examined the association of non‐validated SNPs from the discovery GWAS with prostate cancer risk, progression and mortality.

## Discussion

We found that variants that had been identified in a GWAS^32^ and others linked to them, were the strongest instruments for the exposures examined, as expected. Surprisingly, in ProtecT most of these variants were strong instruments for a related exposure (*i.e.,* IGF‐II) and not for the exposure for which they were originally described (*i.e.,* IGF‐I and IGFBP‐3). The discovery GWAS did not analyse IGF‐II or other IGBP proteins besides IGFBP‐3, which the authors considered a limitation of their study. Additionally, all the variants that proved to be strong instruments for serum IGFs were located on chromosome 7p12.3 in the *IGFBP1/IGFBP3* gene region. This is consistent with the dominant effect of IGFBP‐3 on circulating IGF levels. The IGFs are not stored in any tissue but are constitutively secreted from most tissues and stored in a circulating reservoir by forming a ternary complex with IGFBP‐3 and an acid labile subunit that extends the circulating half‐life of IGFs from 8–12 minutes to 15–18 hr.^33^

To investigate the discrepancy between our findings in ProtecT and the literature reports, we ran an analysis of SNPs confirmed as strong instruments in ProtecT, in ALSPAC mothers (*N* ∼ 400) and children (*N* ∼ 160–450) who had IGF‐I, IGF‐II and IGFBP‐3 measured, and in ∼9,000 men and women from the UKHLS with measures of circulating IGF‐I. Robust associations of *IGFBP1/IGFBP‐3* SNPs with IGF‐II as well as with IGFBP‐3 levels were identified in pregnant women and in children across several ages. None of the SNPs were associated with IGF‐I in ALSPAC. However, in UKHLS the majority of these variants showed an association with IGF‐I concentration, the most convincing being rs700752.

The remaining GWAS‐identified IGF‐associated variants on chromosomes 4p16.1, 6q21, 7p21.3, 10q22.1 and 16p13.3 were not strong instruments in ProtecT, ALSPAC or UKHLS.

When examined in relation to prostate cancer, the validated IGF instruments showed weak associations with Gleason grade. The strongest instrument in the literature and in ProtecT, rs11977526 and other SNPs in LD with it were associated with high (*vs*. low) grade disease. In addition, a few of the strong instruments validated in this study were associated with all‐cause mortality under a non‐additive genetic model (on the basis on an earlier report of non‐additivity in the relationship of rs11977526 and longevity^34^). On the other hand, rs700752 exhibited the strongest association with prostate cancer‐specific mortality under an additive model.

The non‐validated instruments from the discovery GWAS^32^ did not show an association with any prostate cancer outcome, except for rs2153960, which was associated with aggressiveness and mortality. This SNP lies in the *FOXO3* gene, well‐known for its relationship with longevity,^35^ and it is possible that this is driving the association with cancer.

A deeper look into the *IGFBP1/IGFBP3* region revealed at least two independent signals of association with prostate cancer following the regional LD structure (excluding rs700752): one toward the *IGFBP1* gene, and one encompassing the *IGFBP3* gene. The lack of –or marginal‐ association with IGF‐I, IGF‐II and IGFBP‐3 levels of SNPs in or near *IGFBP1* may mean that these variants are predominantly influencing IGFBP‐1 levels. Recently higher circulating IGFBP‐1 was found to be associated with lower prostate cancer risk.^4^, ^36^ It is also conceivable that these signals may all be linked to another, causal signal in the region.

An MR analysis using rs11977526 as the IV, revealed that a large increase in the concentration of IGF‐II or IGFBP‐3 (∼1 SD) would increase the likelihood of progression to high grade cancer by approximately 15%, whilst a similar increase in IGF‐I levels would be protective against disease progression. Conversely, if rs700752 (a SNP not in LD with, and quite distant from rs11977526) is used as an instrument for IGF levels, a one SD increase in IGF‐I or IGFBP‐3 would reduce the risk of prostate cancer‐specific mortality between ∼25% and 50%, depending on the genotype‐exposure estimates considered. Given the association of each SNP with multiple IGF biomarkers the estimates obtained using different sets of instruments and exposures could provide fairly different answers.

In summary, we have confirmed the association of genetic variants that lie toward the *IGFBP3* end of the *IGFBP1/IGFBP3* region with IGFBP‐3 and IGF‐I levels, and we have discovered a novel association of some of the same variants with circulating IGF‐II, which was observed in both ProtecT and ALSPAC. The differences found in the associations of the polymorphisms with the biomarkers could relate to the cohort composition (for instance, differing age structure or sex proportion), the method of assaying blood concentrations (*e.g.,* physical *vs*. chemical dissociation of IGF‐I from IGFBPs used in ProtecT/ALSPAC and UKHLS, respectively) or to having reduced statistical power to detect them, as ProtecT and ALSPAC had low numbers of participants with IGF measures.

Our findings have important implications for MR as the SNPs examined have pleiotropic effects on IGF peptides and it will not be possible to isolate the effect of any one biomarker on an outcome of interest using these instruments. Nevertheless, these variants could be used as strong instruments for the more general causal involvement of the IGF axis on a particular trait or disease, which undoubtedly provides valuable information regarding the mechanisms leading to the onset and progression of the condition. Because of the regional pattern of LD and the lack of data on low frequency variants in *IGFBP1/IGFBP3* it has not been possible to fully identify the functional polymorphisms responsible for variation in IGF levels, which could have helped better define the instruments for MR. In the future a GWAS on circulating IGFBP‐1 might provide useful instruments for this exposure as well.

We have also detected associations of SNPs in *IGFBP‐1/IGBP‐3* with prostate cancer aggressiveness which suggest a positive relationship with higher circulating IGF‐II and possibly IGFBP‐3 (this varies depending on the instrument used). On the other hand, results obtained with instruments rs11977526 and rs700752 independently indicate an inverse association of IGF‐I levels with Gleason grade and mortality. Although these associations were not very strong it is likely that local IGF levels in the prostate may be more prominent and there may be other determinants of such local levels. It is important to replicate of our findings in a non‐overlapping prostate cancer set or using stronger instruments when they become available. Additionally, the association with mortality deserves further scrutiny including a more thorough assessment of the underlying genetic model.

#### Comparison with existing literature on IGF and prostate cancer

Prior studies that have examined the relationship between genetic variants in IGF pathway genes (primarily *IGF1* and *IGFBP3*) and prostate cancer, some of which also analysed circulating IGF proteins, reported for the most part an association of *IGF1* genetic polymorphisms with disease in Europeans, African Americans, Japanese and Chinese.^37^, ^38^, ^39^, ^40^, ^41^, ^42^ Two studies, carried out in African American and Korean men, respectively, showed an association of the *IGFBP3* SNP rs2854744 with IGFBP‐3 levels and prostate cancer risk.^43^, ^44^ Among the studies conducted in European populations that measured circulating IGF‐I and IGFBP‐3, some found an association of the SNPs with serum levels but not with prostate cancer, and of serum levels with prostate cancer.^37^, ^45^, ^46^ Some did not find an association of the SNPs with serum levels, although both the SNPs and the serum levels were associated with prostate cancer,^37^, ^39^ and one identified an association of the genetic variants with serum levels but no association of variants or levels with prostate cancer.^39^

Compared to these studies (with samples sizes ranging from 130 to ∼6,000 patients and an equivalent number of controls), our study had good power, from a large sample size in PRACTICAL, to accurately estimate the genotype‐outcome associations, and obtain precise causal odds ratios.^47^

A number of observational studies have consistently reported positive associations of circulating IGF‐I with prostate cancer, but inferences of causality are limited with observational studies.^3^, ^4^, ^36^ MR is designed to overcome these problems if the exposure is adequately instrumented. Our MR estimates with independent instruments rs11977526 and rs700752 seem to contradict observational studies on the effect of IGF‐I on prostate cancer; however replication with, ideally, non‐pleiotropic instruments is necessary. Observational findings for IGFBP‐3 have been inconsistent,^3^, ^5^, ^6^ whereas IGF‐II and IGFBP‐2 have been investigated less frequently.^3^, ^4^ Regarding IGFBP‐3, results based on the strongest instrument (rs11977526) are concordant with the positive association described in the observational literature^4^, ^5^; however, using another instrument, such as rs700752, suggests a protective effect. Alternatively, assuming our results represent the effect of IGF‐II on disease, they are in agreement with previous findings with respect to PSA‐detected prostate cancer, although they found no evidence for an association of this biomarker with cancer grade.^4^, ^5^

## Conclusions

Using MR to establish the causal effects of a modifiable exposure, such as IGF levels, on an outcome of interest requires genetic variants that qualify as instruments for the exposure given a set of assumptions. Thus, it is important that strong instruments are valid across populations, particularly as two‐sample MR becomes more common. When phenotypes are known to vary significantly with population characteristics it would be desirable to make sure that they are being properly instrumented before engaging in an MR analysis. We have found evidence that the IGF axis contributes to some extent to prostate cancer progression to high grade cancer and mortality but the instruments currently available for circulating IGFs do not allow us to pinpoint which biomarker or biomarkers underlie the causal relationship.

## Supporting information

Supporting Information
For information on how to submit an application for gaining access to EPIC data and/or biospecimens, please follow the instructions at http://epic.iarc.fr/access/index.php, http://www.metadac.ac.uk/data-access-through-metadac/.
