# Supplementary material for: Assessing the role of insulin‐like growth factors and binding proteins in prostate cancer using Mendelian randomization: Genetic variants as instruments for circulating levels
Source: Int J Cancer. 2016 Jun 23;139(7):1520–33. doi: 10.1002/ijc.30206 (PMC4957617; doi:10.1002/ijc.30206)
Supplement: Supplementary file 1 — Supporting Information For information on how to submit an application for gaining access to EPIC data and/or biospecimens, please follow the instructions at http://epic.iarc.fr/access/index.php, http://www.metadac.ac.uk/data-access-through-metadac/. [file IJC-139-1520-s001.docx]

**Supplementary Results**

**Association of SNPs in *IGFBP1/IGFBP3* with prostate cancer risk and progression**

There were a few extra genetic variants showing weak associations with prostate cancer spanning a region of approximately 110 kbs in *IGFBP1/IGFBP3* (from ~45,860 to ~45,970 kbs), besides those reported in the main text (**Supplementary Table 7**, **Supplementary Figure 1**).

Rs2453840 was associated with prostate cancer risk (OR per T allele 0.97; 95% CI 0.95, 0.99) but was either not or weakly associated with IGF levels in ProtecT and ALSPAC, respectively, and was out of HWE in UKHLS (not shown). This SNP was not in LD with rs11977526 (r^2^ = 0.03). A group of three variants in strong LD (rs12671457, rs12671484 and rs2270628), yet not linked to rs2453840 (r^2^ = 0.05 – 0.08) or rs11977526 (r^2^ = 0.005 – 0.01) (**Supplementary Figure 1**), was also associated with prostate cancer risk (**Supplementary Table 7**), however exhibited weak, inconsistent associations with IGF levels in ProtecT, ALSPAC and UKHLS (not shown).

The analysis of Gleason grade (< 7 (reference) vs ≥ 7) yielded the most robust association with rs2949833 (OR per T allele 1.08; 95% CI 1.02, 1.12) (**Supplementary Table 7**). In a sensitivity analysis where Gleason grade was categorised as <8 (reference)/≥ 8^1^ this association was no longer evident, whereas SNP rs12702181 showed a consistent association with both cut-offs (ORs per G allele 0.95; 95% CI 0.92, 0.99, and 0.93; 95% CI 0.89, 0.98, respectively). Additionally, the analysis of disease stage (localised (reference) vs advanced) identified an association with rs12666800 only (OR per A allele 0.90; 95% CI 0.85, 0.96) (**Supplementary Table 7**). Rs2949833 was strongly associated with IGF-II in ProtecT controls, with IGF-II and IGFBP-3 in ALSPAC, and with IGF-I in UKHLS (not shown), and is in LD with rs11977526 (r^2^ = 0.69). Its T allele increases serum IGF-II and IGFBP-3 and decreases IGF-I. Rs12666800 and rs12702181 were not associated with IGF levels (not shown) and are not in strong LD with any of the above markers (**Supplementary Figure 1**).

**Association of SNPs in *IGFBP1/IGFBP3* with all-cause and prostate cancer-specific mortality**

Genetic variants that were associated with prostate cancer risk showed evidence of association with 15-year all-cause and prostate cancer-specific mortality (rs12671457, rs12671484, rs2270628), or just with mortality due to prostate cancer (rs2453840), based on an additive model (**Supplementary Table 5**). Of those associated with prostate cancer grade and stage, rs2949833 and rs12702181 were associated with both all-cause and prostate cancer-specific mortality following a non-additive and an additive pattern, respectively, whilst rs12666800 was not associated with mortality at all.

**Association of non-validated SNPs from the discovery GWAS with prostate cancer risk, progression and mortality**

SNPs found in the discovery GWAS of IGF-I and IGFBP-3 levels^2^, which were marginally associated with IGF biomarkers in ProtecT and the replication cohorts (**Supplementary Table 4**), were not associated with prostate cancer, with the exception of rs2153960. This variant showed a strong association with stage of disease (localised vs advanced OR per A allele 0.93; 95% CI 0.89, 0.98) and a weaker (and on the opposite direction) association with cancer grade (low vs high OR per A allele 1.05; 95% CI 1.01, 1.11) (**Supplementary Table 8**). Rs2153960 was also associated with all-cause mortality (HR per A allele 0.93; 95% CI 0.89, 0.97) more strongly than with prostate cancer-specific mortality (HR per A allele 0.95; 95% CI 0.88, 1.02) (non-proportional hazards, p < 0.05). The A allele was found to increase IGF-I levels in the GWAS.

**Supplementary References**

1. Tefilli M V., Gheiler EL, Tiguert R, Sakr W, Grignon DJ, Banerjee M, Pontes JE, Wood DP. Should Gleason score 7 prostate cancer be considered a unique grade category? *Urology* 1999;53:372–7.

2. Kaplan RC, Petersen A-K, Chen M-H, Teumer A, Glazer NL, Döring A, Lam CSP, Friedrich N, Newman A, Müller M, Yang Q, Homuth G, et al. A genome-wide association study identifies novel loci associated with circulating IGF-I and IGFBP-3. *Hum Mol Genet* 2011;20:1241–51.

3. Gu F, Schumacher FR, Canzian F, Allen NE, Albanes D, Berg CD, Berndt SI, Boeing H, Bueno-de-Mesquita HB, Buring JE, Chabbert-Buffet N, Chanock SJ, et al. Eighteen insulin-like growth factor pathway genes, circulating levels of IGF-I and its binding protein, and risk of prostate and breast cancer. *Cancer Epidemiol Biomarkers Prev* 2010;19:2877–87.

4. Canzian F, McKay JD, Cleveland RJ, Dossus L, Biessy C, Boillot C, Rinaldi S, Llewellyn M, Chajes V, Clavel-Chapelon F, Tehard B, Chang-Claude J, et al. Genetic variation in the growth hormone synthesis pathway in relation to circulating Insulin-Like Growth Factor-I, Insulin-Like Growth Factor Binding Protein-3, and breast cancer risk: Results from the European Prospective Investigation into Cancer and Nu. *Cancer Epidemiol Biomarkers Prev* 2005;14:2316–25.

5. Mong JLY, Ng MCY, Guldan GS, Tam CHT, Lee HM, Ma RCW, So WY, Wong GWK, Kong APS, Chan JCN, Waye MMY. Associations of the growth hormone receptor (GHR) gene polymorphisms with adiposity and IGF-I activity in adolescents. *Clin Endocrinol (Oxf)* 2010;73:313–22.

6. Biong M, Gram IT, Brill I, Johansen F, Solvang HK, Alnaes GIG, Fagerheim T, Bremnes Y, Chanock SJ, Burdett L, Yeager M, Ursin G, et al. Genotypes and haplotypes in the insulin-like growth factors, their receptors and binding proteins in relation to plasma metabolic levels and mammographic density. *BMC Med Genomics* 2010;3:9.

7. Taverne CW, Verheus M, McKay JD, Kaaks R, Canzian F, Grobbee DE, Peeters PHM, van Gils CH. Common genetic variation of insulin-like growth factor-binding protein 1 (IGFBP-1), IGFBP-3, and acid labile subunit in relation to serum IGF-I levels and mammographic density. *Breast Cancer Res Treat* 2010;1.

8. Schumacher FR, Cheng I, Freedman ML, Mucci L, Allen NE, Pollak MN, Hayes RB, Stram DO, Canzian F, Henderson BE, Hunter DJ, Virtamo J, et al. A comprehensive analysis of common IGF1, IGFBP1 and IGFBP3 genetic variation with prospective IGF-I and IGFBP-3 blood levels and prostate cancer risk among Caucasians. *Hum Mol Genet* 2010;19:3089–101.

9. Patel A V, Cheng I, Canzian F, Le Marchand L, Thun MJ, Berg CD, Buring J, Calle EE, Chanock S, Clavel-Chapelon F, Cox DG, Dorronsoro M, et al. IGF-1, IGFBP-1, and IGFBP-3 polymorphisms predict circulating IGF levels but not breast cancer risk: findings from the Breast and Prostate Cancer Cohort Consortium (BPC3). *PLoS One* 2008;3:e2578.

10. D’Aloisio AA, Schroeder JC, North KE, Poole C, West SL, Travlos GS, Baird DD. IGF-I and IGFBP-3 polymorphisms in relation to circulating levels among African American and Caucasian women. *Cancer Epidemiol Biomarkers Prev* 2009;18:954–66.

11. Canzian F, McKay JD, Cleveland RJ, Dossus L, Biessy C, Rinaldi S, Landi S, Boillot C, Monnier S, Chajès V, Clavel-Chapelon F, Téhard B, et al. Polymorphisms of genes coding for insulin-like growth factor 1 and its major binding proteins, circulating levels of IGF-I and IGFBP-3 and breast cancer risk: results from the EPIC study. *Br J Cancer* 2006;94:299–307.

12. Petry CJ, Ong KK, Barratt BJ, Wingate D, Cordell HJ, Ring SM, Pembrey ME, Reik W, Todd JA, Dunger DB. Common polymorphism in H19 associated with birthweight and cord blood IGF-II levels in humans. *BMC Genet* 2005;6:22.

13. Al-Zahrani A, Sandhu MS, Luben RN, Thompson D, Baynes C, Pooley KA, Luccarini C, Munday H, Perkins B, Smith P, Pharoah PDP, Wareham NJ, et al. IGF1 and IGFBP3 tagging polymorphisms are associated with circulating levels of IGF1, IGFBP3 and risk of breast cancer. *Hum Mol Genet* 2006;15:1–10.

14. Verheus M, Maskarinec G, Woolcott CG, Haiman CA, Le Marchand L, Henderson BE, Cheng I, Kolonel LN. IGF1, IGFBP1, and IGFBP3 genes and mammographic density: The Multiethnic Cohort. *Int J Cancer* 2009;127:1115–23.

15. Vargas T, Martinez-Garcia A, Antequera D, Vilella E, Clarimon J, Mateo I, Sanchez-Juan P, Rodriguez-Rodriguez E, Frank A, Rosich-Estrago M, Lleo A, Molina-Porcel L, et al. IGF-I gene variability is associated with an increased risk for AD. *Neurobiol Aging* 2011;32:556.e3–556.e11.

16. Franco L, Williams FMK, Trofimov S, Malkin I, Surdulescu G, Spector T, Livshits G. Assessment of age-related changes in heritability and IGF-1 gene effect on circulating IGF-1 levels. *Age (Omaha)* 2014;36:1443–52.

17. Rzehak P, Grote V, Lattka E, Weber M, Gruszfeld D, Socha P, Closa-Monasterolo R, Escribano J, Giovannini M, Verduci E, Goyens P, Martin F, et al. Associations of IGF-1 gene variants and milk protein intake with IGF-I concentrations in infants at age 6 months - results from a randomized clinical trial. *Growth Horm IGF Res* 2013;23:149–58.

18. Terry KL, Tworoger SS, Gates MA, Cramer DW, Hankinson SE. Common genetic variation in IGF1, IGFBP1 and IGFBP3 and ovarian cancer risk. *Carcinogenesis* 2009;30:2042–6.

19. Palles C, Johnson N, Coupland B, Taylor C, Carvajal J, Holly J, Fentiman IS, Silva IDS, Ashworth A, Peto J, Fletcher O. Identification of genetic variants that influence circulating IGF1 levels: a targeted search strategy. *Hum Mol Genet* 2008;17:1457–64.

**Supplementary Table 1. Published SNPs associated with IGF levels tested in ProtecT controls.**

| **SNP** | **nearest gene** | **chromosome** | **position** | **function** | **effect allele/non-effect allele^a^** | **effect allele frequency** | **published association** | **reference** |
| --- | --- | --- | --- | --- | --- | --- | --- | --- |
| rs3770473 | IGFBP2/5 | 2q33-q34 | 216634116 | intronic | G/T | 0.291 | IGF-I/IGFBP3 | ^3^ |
| rs300982 | POU1F1 | 3p11 | 87276603 | 5'upstream | A/G | 0.042 | IGFBP3 | ^4^ |
| **rs4234798** | SORCS2 | 4p16.1 | 7218206 | intronic | G/T | 0.616 | IGFBP3 | ^2^ |
| rs7703713 | GHR | 5p13-p12 | 42555829 | intronic | A/G | 0.254 | IGF-I | ^5^ |
| **rs2153960** | FOXO3 | 6q21 | 108666981 | intronic | A/G | 0.714 | IGF-I | ^2^ |
| rs998075 | IGF2R | 6q26 | 160047246 | coding (T713T) | G/A | 0.456 | IGF-I | ^6^ |
| rs998074 | IGF2R | 6q26 | 160047351 | intronic | C/T | 0.456 | IGF-I | ^6^ |
| **rs7780564** | RPA3-AS1 | 7p21.3 | 7843692 | intronic | C/A | 0.539 | IGF-I | ^2^ |
| rs10228265 | IGFBP1 | 7p12.3 | 45869316 |  | A/G | 0.686 | IGFBP3 | ^3^ |
| rs1908751 | IGFBP1 | 7p12.3 | 45895920 |  | T/C | 0.264 | IGF-I | ^7^ |
| rs2270628 | IGFBP3 | 7p12.3 | 45909971 |  | C/T | 0.791 | IGFBP3 | ^3,8^ |
| rs6670 | IGFBP3 | 7p12.3 | 45912655 | 3'UTR | A/T | 0.795 | IGF-I | ^9^ |
| rs3110697 | IGFBP3 | 7p12.3 | 45915430 | intronic | G/A | 0.580 | IGFBP3 | ^3,8^ |
| rs9282734 | IGFBP3 | 7p12.3 | 45917370 | coding (H158P) | G/T | 0.004 | IGFBP3 | ^10^ |
| rs2471551 | IGFBP3 | 7p12.3 | 45917456 | intronic | G/C | 0.809 | IGFBP3 | ^11^ |
| rs2132572 | IGFBP3 | 7p12.3 | 45921946 | 5'upstream | C/T | 0.771 | IGFBP3/IGF-I | ^11^ |
| rs2132571 | IGFBP3 | 7p12.3 | 45922075 | 5'upstream | C/T | 0.688 | IGFBP3 | ^11^ |
| rs924140 | IGFBP3 | 7p12.3 | 45923515 |  | T/C | 0.536 | IGFBP3 | ^10^ |
| **rs1496499** | IGFBP3 | 7p12.3 | 45939424 |  | G/T | 0.459 | IGF-I^b^ | ^2^ |
| **rs11977526** | IGFBP3 | 7p12.3 | 45968511 |  | A/G | 0.401 | IGFBP3/IGF-I^b^ | ^2^ |
| **rs700752** | IGFBP3 | 7p12.3 | 46713955 |  | G/C | 0.650 | IGF-I/IGFBP3 | ^2^ |
| **rs1245541** | SPOCK2 | 10q22.1 | 72089881 | 5'upstream | G/A | 0.600 | IGF-I | ^2^ |
| rs217727 | H19/MIR675 | 11p15.5 | 1995678 | 3'downstream | A/G | 0.201 | IGF2 | ^12^ |
| rs6214 | IGF1 | 12q23.2 | 102399791 | 3'UTR | T/C | 0.426 | IGF-I | ^10^ |
| rs1520220 | IGF1 | 12q23.2 | 102402744 |  | G/C | 0.164 | IGF-I | ^3^ |
| rs5742694 | IGF1 | 12q23.2 | 102405458 | intronic | A/C | 0.778 | IGF-I | ^13^ |
| rs978458 | IGF1 | 12q23.2 | 102408461 | intronic | T/C | 0.233 | IGF-I | ^14^ |
| rs5742678 | IGF1 | 12q23.2 | 102420554 | intronic | C/G | 0.232 | IGF-I | ^13^ |
| rs972936 | IGF1 | 12q23.2 | 102431143 | intronic | C/T | 0.767 | IGF-I | ^15^ |
| rs2288378 | IGF1 | 12q23.2 | 102436230 | intronic | T/C | 0.221 | IGF-I | ^16^ |
| rs7136446 | IGF1 | 12q23.2 | 102444737 | intronic | C/T | 0.417 | IGF-I | ^3^ |
| rs10735380 | IGF1 | 12q23.2 | 102450458 | intronic | G/A | 0.269 | IGF-I | ^3^ |
| rs2195239 | IGF1 | 12q23.2 | 102462924 | intronic | G/C | 0.219 | IGF-I | ^17^ |
| rs12821878 | IGF1 | 12q23.2 | 102473889 | intronic | G/A | 0.740 | IGF-I | ^8^ |
| rs5742615 | IGF1 | 12q23.2 | 102477023 | intronic | T/G | 0.009 | IGF-I | ^13^ |
| rs2162679 | IGF1 | 12q23.2 | 102477481 | intronic | T/C | 0.854 | IGFBP3 | ^11^ |
| rs5742612 | IGF1 | 12q23.2 | 102481086 | 5'upstream | G/A | 0.031 | IGFBP3 | ^10^ |
| rs35767 | IGF1 | 12q23.2 | 102481791 | 5'upstream | A/G | 0.147 | IGF-I | ^8^ |
| rs35766 | IGF1 | 12q23.2 | 102486695 | promoter | C/T | 0.146 | IGF-I | ^16^ |
| rs35765 | IGF1 | 12q23.2 | 102487918 | promoter | T/G | 0.113 | IGF-I | ^11^ |
| rs7965399 | IGF1 | 12q23.2 | 102497908 |  | C/T | 0.039 | IGF-I | ^9^ |
| rs11111285 | IGF1 | 12q23.2 | 102501478 |  | G/A | 0.039 | IGF-I/IGFBP2 | ^18^ |
| rs855211 | IGF1 | 12q23.2 | 102517032 |  | A/G | 0.150 | IGF-I | ^16^ |
| rs10778177 | IGF1 | 12q23.2 | 102543168 |  | C/T | 0.147 | IGF-I | ^19^ |
| rs855203 | IGF1 | 12q23.2 | 102564295 |  | C/A | 0.090 | IGF-I | ^16^ |
| rs1457596 | IGF1 | 12q23.2 | 102568729 |  | A/G | 0.088 | IGF-I | ^16^ |
| rs7964748 | IGF1 | 12q23.2 | 102571551 |  | A/G | 0.810 | IGF-I | ^16^ |
| rs907806 | IGF1R | 15q25-q26 | 98801652 | intronic | G/A | 0.114 | IGFBP3 | ^6^ |
| rs213656 | SSTR5 | 16p13.3 | 1063847 | 3'downstream | T/G | 0.398 | IGF-I | ^3^ |
| rs3751830 | SSTR5 | 16p13.3 | 1078406 | 5'upstream | C/T | 0.570 | IGF-I | ^3^ |
| rs197056 | SSTR5 | 16p13.3 | 1081695 | 3'downstream | A/G | 0.379 | IGF-I | ^3^ |
| rs174643 | SSTR5 | 16p13.3 | 1083049 | 3'downstream | G/A | 0.442 | IGF-I | ^3^ |
| rs1178436 | NUBP2 | 16p13.3 | 1781652 | 5'upstream | C/T | 0.810 | IGFBP3 | ^3^ |
| **rs1065656** | IGFALS/NUBP2 | 16p13.3 | 1788835 | coding (P164R) | G/C | 0.696 | IGFBP3 | ^2^ |
| rs17559 | IGFALS/NUBP2 | 16p13.3 | 1791032 | coding (Y500Y) | A/G | 0.105 | IGFBP3 | ^3^ |
| rs11865665 | HAGH | 16p13.3 | 1815535 | intronic | G/A | 0.074 | IGFBP3 | ^3^ |

GRCh38.p2. In bold, SNPs uncovered in a GWAS of IGF-I and IGFBP-3 levels^2^.

^a^The effect allele increases IGF levels.

^b^Associated with IGF-I after adjustment for IGFBP-3.

**Supplementary Table 2. IGF biomarker levels in ProtecT, ALSPAC and UKHLS.**

| **biomarker** | **study** | **population** | **mean (ng/ml)** | **SD** | **N** |
| --- | --- | --- | --- | --- | --- |
| IGF-I | ProtecT | men | 163.2 | 57.0 | 727 |
| IGF-I | ALSPAC | pregnant women | 191.1 | 54.4 | 375 |
| IGF-I | ALSPAC | cord blood | 96.5 | 37.7 | 494 |
| IGF-I | ALSPAC | children ~61 months old | 161.1 | 68.3 | 438 |
| IGF-I | ALSPAC | children ~7 years old | 143.9 | 50.2 | 380 |
| IGF-I | ALSPAC | children ~8 years old | 160.4 | 57.3 | 355 |
| IGF-I | UKHLS | men | 137.7 | 47.4 | 3987 |
| IGF-I | UKHLS | women | 133.9 | 52.0 | 5084 |
|  |  |  |  |  |  |
| IGF-II | ProtecT | men | 733.7 | 265.3 | 718 |
| IGF-II | ALSPAC | pregnant women | 627.9 | 201.4 | 365 |
| IGF-II | ALSPAC | cord blood | 264.0 | 80.0 | 495 |
| IGF-II | ALSPAC | children ~61 months old | 398.8 | 104.7 | 438 |
| IGF-II | ALSPAC | children ~7 years old | 476.3 | 108.1 | 166 |
| IGF-II | ALSPAC | children ~8 years old | n/a | n/a | n/a |
|  |  |  |  |  |  |
| IGFBP-3 | ProtecT | men | 4370.8 | 1055.7 | 712 |
| IGFBP-3 | ALSPAC | pregnant women | 5379.9 | 1354.6 | 375 |
| IGFBP-3 | ALSPAC | cord blood | 1384.6 | 491.6 | 147 |
| IGFBP-3 | ALSPAC | children ~61 months old | n/a | n/a | n/a |
| IGFBP-3 | ALSPAC | children ~7 years old | 3621.2 | 1049.9 | 380 |
| IGFBP-3 | ALSPAC | children ~8 years old | 5621.7 | 1758.7 | 355 |
|  |  |  |  |  |  |
| IGFBP-2 | ProtecT | men | 726.2 | 444.7 | 724 |

**Supplementary Table 3. Association of SNPs validated as strong instruments with covariables in ProtecT controls.**

| **SNP** | **effect allele/other allele^a^** | **beta/OR^b^** | **95% CI** | **p-value** |
| --- | --- | --- | --- | --- |
| **age** (years, N=728) |  |  |  |  |
| rs11977526 | A/G | 0.56 | (0.02,1.11) | 0.04 |
| rs1496499 | G/T | 0.50 | (-0.02,1.02) | 0.06 |
| rs2132571 | C/T | 0.21 | (-0.34,0.76) | 0.45 |
| rs3110697 | G/A | 0.36 | (-0.17,0.88) | 0.18 |
| rs700752 | G/C | 0.43 | (-0.10,0.95) | 0.11 |
| rs924140 | T/C | 0.47 | (-0.05,0.99) | 0.08 |
| **BMI** (kg/m^2^, N=456) |  |  |  |  |
| rs11977526 | A/G | -0.22 | (-0.78,0.35) | 0.45 |
| rs1496499 | G/T | -0.31 | (-0.84,0.23) | 0.26 |
| rs2132571 | C/T | 0.08 | (-0.48,0.64) | 0.79 |
| rs3110697 | G/A | 0.00 | (-0.54,0.55) | 0.99 |
| rs700752 | G/C | 0.47 | (-0.07,1.00) | 0.09 |
| rs924140 | T/C | -0.31 | (-0.84,0.23) | 0.26 |
| **height** (cm, N=456) |  |  |  |  |
| rs11977526 | A/G | -0.53 | (-1.53,0.47) | 0.30 |
| rs1496499 | G/T | -0.28 | (-1.23,0.66) | 0.56 |
| rs2132571 | C/T | -0.67 | (-1.65,0.31) | 0.18 |
| rs3110697 | G/A | 0.02 | (-0.94,0.98) | 0.96 |
| rs700752 | G/C | -0.63 | (-1.58,0.32) | 0.19 |
| rs924140 | T/C | -0.28 | (-1.23,0.66) | 0.56 |
| **leg length** (cm, N=447) |  |  |  |  |
| rs11977526 | A/G | -0.10 | (-0.74,0.53) | 0.75 |
| rs1496499 | G/T | 0.04 | (-0.56,0.65) | 0.89 |
| rs2132571 | C/T | -0.20 | (-0.84,0.43) | 0.53 |
| rs3110697 | G/A | 0.20 | (-0.42,0.81) | 0.53 |
| rs700752 | G/C | -0.29 | (-0.90,0.32) | 0.35 |
| rs924140 | T/C | 0.04 | (-0.56,0.65) | 0.89 |
| **PSA** (ng/ml, N=728)^c^ |  |  |  |  |
| rs11977526 | A/G | -0.10 | (-0.18,-0.01) | 0.02 |
| rs1496499 | G/T | -0.04 | (-0.13,0.04) | 0.28 |
| rs2132571 | C/T | 0.004 | (-0.08,0.09) | 0.93 |
| rs3110697 | G/A | -0.04 | (-0.12,0.04) | 0.32 |
| rs700752 | G/C | 0.04 | (-0.04,0.12) | 0.33 |
| rs924140 | T/C | -0.04 | (-0.12,0.04) | 0.30 |
| **BPH** (0=no/1=possible&yes, N=711) |  |  |  |  |
| rs11977526 | A/G | 0.75 | (0.52,1.09) | 0.13 |
| rs1496499 | G/T | 0.68 | (0.48,0.97) | 0.03 |
| rs2132571 | C/T | 0.66 | (0.46,0.93) | 0.02 |
| rs3110697 | G/A | 0.83 | (0.58,1.16) | 0.28 |
| rs700752 | G/C | 0.87 | (0.61,1.23) | 0.43 |
| rs924140 | T/C | 0.68 | (0.48,0.97) | 0.03 |
| **diabetes** (0=no/1=yes, N=427) |  |  |  |  |
| rs11977526 | A/G | 0.99 | (0.59,1.66) | 0.98 |
| rs1496499 | G/T | 0.99 | (0.60,1.62) | 0.96 |
| rs2132571 | C/T | 0.91 | (0.55,1.51) | 0.72 |
| rs3110697 | G/A | 1.01 | (0.61,1.69) | 0.96 |
| rs700752 | G/C | 1.35 | (0.80,2.27) | 0.27 |
| rs924140 | T/C | 0.99 | (0.61,1.61) | 0.96 |

^a^The effect allele increases IGF levels.

^b^Beta coefficient for continuous variables, OR for categorical variables.

^c^PSA was natural log transformed.

All regression models were adjusted for age (when appropriate) and 10 principal components.

**Supplementary Table 4. SNPs validated in ProtecT and IGF levels in ALSPAC and UKHLS participants.**

| **SNP** | **effect allele/other allele^a^** | **change in serum IGF/allele^b^** | **95% CI** | **p-value** | **R^2^(%)^c^** | **F^c^** |
| --- | --- | --- | --- | --- | --- | --- |
| **mothers** |  |  |  |  |  |  |
| **IGF-I** (ng/ml, N=375) |  |  |  |  |  |  |
| rs700752 | G/C | 1.61 | (-6.40,9.62) | 0.69 | 0.0 | 0.01 |
| **IGF-II** (ng/ml, N=365) |  |  |  |  |  |  |
| rs11977526 | A/G | 0.08 | (0.04,0.12) | 3.21x10^-4^ | 4.3 | 17.9 |
| rs1496499 | G/T | 0.08 | (0.04,0.13) | 1.52x10^-4^ | 4.1 | 17.1 |
| rs2132571 | C/T | 0.07 | (0.03,0.12) | 0.001 | 2.8 | 11.4 |
| rs2854744 | A/C | 0.08 | (0.04,0.13) | 1.52x10^-4^ | 1.6 | 6.7 |
| rs2854746 | C/G | 0.09 | (0.05,0.14) | 2.76x10^-5^ | 2.8 | 12.4 |
| rs3110697 | G/A | 0.05 | (0.01,0.09) | 0.029 | 1.3 | 5.4 |
| rs924140 | T/C | 0.08 | (0.04,0.13) | 1.52x10^-4^ | 4.2 | 17.5 |
| **IGFBP-3** (ng/ml, N=375) |  |  |  |  |  |  |
| rs11977526 | A/G | 339.39 | (143.10, 535.69) | 0.001 | 2.8 | 12.4 |
| rs1496499 | G/T | 265.22 | (72.10,458.35) | 0.01 | 1.6 | 6.7 |
| rs2132571 | C/T | 224.61 | (23.19,426.04) | 0.03 | 0.8 | 3.5 |
| rs2854744 | A/C | 265.22 | (72.10,458.35) | 0.01 | 4.1 | 17.1 |
| rs2854746 | C/G | 336.08 | (141.56, 530.60) | 0.001 | 4.9 | 20.4 |
| rs3110697 | G/A | 207.94 | (9.67,406.22) | 0.04 | 1.1 | 4.9 |
| rs700752 | G/C | 107.98 | (-89.30,305.25) | 0.28 | 0.3 | 1.2 |
| rs924140 | T/C | 265.22 | (72.10,458.35) | 0.01 | 1.5 | 6.3 |
|  |  |  |  |  |  |  |
| **cord blood** |  |  |  |  |  |  |
| **IGF-I** (ng/ml, N=494) |  |  |  |  |  |  |
| rs700752 | G/C | 1.75 | (-3.04,6.54) | 0.47 | 0.1 | 0.6 |
| **IGF-II** (ng/ml, N=495) |  |  |  |  |  |  |
| rs11977526 | A/G | 15.73 | (5.32,26.14) | 0.003 | 1.6 | 8.4 |
| rs1496499 | G/T | 17.58 | (7.53, 27.63) | 0.001 | 2.3 | 12.2 |
| rs2132571 | C/T | 16.28 | (5.33, 27.23) | 0.004 | 1.5 | 7.7 |
| rs2854744 | A/C | 17.78 | (7.77,27.80) | 0.001 | 2.3 | 12.5 |
| rs2854746 | C/G | 19.59 | (9.21,29.97) | 2.34x10^-4^ | 2.5 | 13.6 |
| rs3110697 | G/A | 9.61 | (-0.64,19.85) | 0.07 | 0.8 | 4.0 |
| rs924140 | T/C | 17.78 | (7.77,27.80) | 0.001 | 2.3 | 12.5 |
| **IGFBP-3** (ng/ml, N=147) |  |  |  |  |  |  |
| rs11977526 | A/G | 39.05 | (-77.07,155.18) | 0.51 | 0.1 | 0.2 |
| rs1496499 | G/T | 33.68 | (-80.37,147.72) | 0.56 | 0.1 | 0.2 |
| rs2132571 | C/T | 85.90 | (-39.13,210.94) | 0.18 | 0.1 | 0.2 |
| rs2854744 | A/C | 33.68 | (-80.37,147.72) | 0.56 | 0.1 | 0.2 |
| rs2854746 | C/G | 42.38 | (-73.86,158.62) | 0.47 | 0.2 | 0.3 |
| rs3110697 | G/A | -36.83 | (-153.32,79.66) | 0.53 | 0.0 | 0.0 |
| rs700752 | G/C | 55.04 | (-64.06,174.14) | 0.36 | 0.3 | 0.5 |
| rs924140 | T/C | 33.68 | (-80.37,147.72) | 0.56 | 0.1 | 0.2 |
|  |  |  |  |  |  |  |
| **children ~61 months old** |  |  |  |  |  |  |
| **IGF-I** (ng/ml, N=438) |  |  |  |  |  |  |
| rs700752 | G/C | 2.62 | (-6.96,12.21) | 0.59 | 0.1 | 0.6 |
| **IGF-II** (ng/ml, N=438) |  |  |  |  |  |  |
| rs11977526 | A/G | 48.09 | (33.73, 62.46) | 1.37x10^-10^ | 9.0 | 46.4 |
| rs1496499 | G/T | 46.96 | (33.11,60.81) | 8.29x10^-11^ | 10.0 | 51.8 |
| rs2132571 | C/T | 28.62 | (13.21,44.03) | 2.95x10^-4^ | 4.2 | 20.4 |
| rs2854744 | A/C | 47.91 | (34.04,61.78) | 3.78x10^-11^ | 10.4 | 53.9 |
| rs2854746 | C/G | 52.11 | (38.07,66.15) | 1.46x10^-12^ | 11.4 | 60.2 |
| rs3110697 | G/A | 39.06 | (24.71,53.42) | 1.46x10^-7^ | 5.0 | 24.7 |
| rs924140 | T/C | 47.91 | (34.04,61.78) | 3.78x10^-11^ | 10.4 | 53.9 |
|  |  |  |  |  |  |  |
| **children ~7 years old** |  |  |  |  |  |  |
| **IGF-I** (ng/ml, N=380) |  |  |  |  |  |  |
| rs700752 | G/C | 5.94 | (-1.53,13.41) | 0.12 | 0.8 | 3.4 |
| **IGF-II** (ng/ml, N=166) |  |  |  |  |  |  |
| rs11977526 | A/G | 31.98 | (8.48,55.49) | 0.01 | 7.6 | 14.8 |
| rs1496499 | G/T | 22.69 | (0.35,45.73) | 0.05 | 3.9 | 7.2 |
| rs2132571 | C/T | 23.17 | (1.36,47.70) | 0.06 | 2.9 | 5.4 |
| rs2854744 | A/C | 22.69 | (0.35,45.73) | 0.05 | 3.9 | 7.2 |
| rs2854746 | C/G | 33.78 | (10.48,57.08) | 0.01 | 8.5 | 16.7 |
| rs3110697 | G/A | 23.33 | (-1.68,48.35) | 0.07 | 2.6 | 4.8 |
| rs924140 | T/C | 22.69 | (0.35,45.73) | 0.05 | 3.9 | 7.2 |
| **IGFBP-3** (ng/ml, N=380) |  |  |  |  |  |  |
| rs11977526 | A/G | 180.13 | (30.55,329.71) | 0.02 | 1.8 | 7.3 |
| rs1496499 | G/T | 183.17 | (34.24,332.10) | 0.02 | 1.6 | 6.7 |
| rs2132571 | C/T | 195.00 | (32.44,357.56) | 0.02 | 1.3 | 5.5 |
| rs2854744 | A/C | 183.17 | (34.24,332.10) | 0.02 | 1.6 | 6.7 |
| rs2854746 | C/G | 197.93 | (49.75,346.11) | 0.01 | 2.2 | 9.1 |
| rs3110697 | G/A | 116.53 | (-41.40,274.45) | 0.15 | 0.4 | 1.8 |
| rs700752 | G/C | 299.97 | (144.71,455.24) | 1.70E-04 | 4.1 | 17.3 |
| rs924140 | T/C | 183.17 | (34.24,332.10) | 0.02 | 1.6 | 6.7 |
|  |  |  |  |  |  |  |
| **children ~8 years old** |  |  |  |  |  |  |
| **IGF-I** (ng/ml, N=355) |  |  |  |  |  |  |
| rs700752 | G/C | 6.94 | (-2.11,15.99) | 0.13 | 1.5 | 5.6 |
| **IGFBP-3** (ng/ml, N=355) |  |  |  |  |  |  |
| rs11977526 | A/G | 518.28 | (264.19,772.36) | 7.40x10^-5^ | 4.2 | 16.6 |
| rs1496499 | G/T | 592.29 | (347.95,836.63) | 2.76x10^-6^ | 6.1 | 24.5 |
| rs2132571 | C/T | 510.06 | (231.11,789.00) | 3.70x10^-4^ | 4.5 | 17.8 |
| rs2854744 | A/C | 583.51 | (337.25,829.77) | 4.53x10^-6^ | 5.9 | 23.9 |
| rs2854746 | C/G | 620.35 | (368.99,871.71) | 1.84x10^-6^ | 6.0 | 24.0 |
| rs3110697 | G/A | 458.17 | (206.94,709.41) | 3.83x10^-4^ | 2.6 | 10.1 |
| rs700752 | G/C | 432.12 | (160.40,703.84) | 0.002 | 2.7 | 10.3 |
| rs924140 | T/C | 581.61 | (334.75,828.48) | 5.11x10^-6^ | 5.9 | 23.6 |
|  |  |  |  |  |  |  |
| **UKHLS** |  |  |  |  |  |  |
| **IGF-I** (ng/ml, N=8700)^d^ |  |  |  |  |  |  |
| rs11977526 | A/G | -0.02 | (-0.03,-0.01) | 2.83x10^-5^ | 0.1 | 12.7 |
| rs1496499 | G/T | -0.02 | (-0.03,-0.01) | 4.96x10^-4^ | 0.1 | 6.0 |
| rs2132571 | C/T | -0.01 | (-0.02,0.002) | 0.14 | 0.01 | 0.8 |
| rs2854744 | A/C | -0.02 | (-0.03,-0.01) | 0.001 | 0.1 | 6.0 |
| rs2854746 | C/G | -0.02 | (-0.03,-0.01) | 4.40x10^-5^ | 0.1 | 11.4 |
| rs3110697 | G/A | -0.01 | (-0.02,-0.003) | 0.009 | 0.04 | 4.0 |
| rs700752 | G/C | 0.03 | (0.02,0.04) | 4.56x10^-10^ | 0.3 | 24.4 |
| rs924140 | T/C | -0.02 | (-0.03,-0.01) | 0.001 | 0.1 | 5.8 |

^a^The effect allele increases IGF levels according to the literature.

^b^Regression models in mothers were adjusted for gestational age and 10 principal components. Regression models in children were adjusted for age at clinic visit, sex and 10 principal components. Regression models in UKHLS were adjusted for age, sex and 20 principal components.

^c^R^2^ and F-statistic from unadjusted linear regression of SNP on IGF concentration.

^d^IGF-1 concentration was natural log-transformed for analysis. Effects given correspond to the change in ln(IGF-I) per allele. In an earlier study there was an inverse association of the alleles that increased serum IGFBP-3 with circulating IGF-I, after adjustment for IGFBP-3 levels^2^, at SNPs rs11977526 and rs1496499. We observed a similar inverse association without adjustment since UKHLKS did not measure IGFBP-3 concentration.

**Supplementary Table 5. SNPs identified in the discovery GWAS but not validated in ProtecT, and IGF levels in ALSPAC and UKHLS participants**

| **SNP** | **effect/non-effect allele^a^** | **change in serum IGF/allele^b^** | **95% CI** | **p-value** | **R^2^(%)^c^** | **F^c^** |
| --- | --- | --- | --- | --- | --- | --- |
| **cord blood** |  |  |  |  |  |  |
| **IGFBP-3** (ng/ml, N=147) |  |  |  |  |  |  |
| rs2153960 | A/G | -129.86 | (-258.45,-1.27) | 0.05 | 1.4 | 2.2 |
|  |  |  |  |  |  |  |
| **children ~ 61 months old** |  |  |  |  |  |  |
| **IGF-II** (ng/ml, N=438) |  |  |  |  |  |  |
| rs1065656 | G/C | 15.34 | (-0.26,30.94) | 0.05 | 0.7 | 3.2 |
| rs4234798 | T/G | 18.90 | (4.56,33.23) | 0.01 | 1.7 | 7.9 |
|  |  |  |  |  |  |  |
| **children ~ 7 years old** |  |  |  |  |  |  |
| **IGFBP-3** (ng/ml, N=380) |  |  |  |  |  |  |
| rs1245541 | G/A | 194.26 | (30.08,358.44) | 0.02 | 1.7 | 6.9 |
| rs4234798 | T/G | 159.72 | (8.34,311.09) | 0.04 | 1.3 | 5.3 |
|  |  |  |  |  |  |  |
| **children ~ 8 years old** |  |  |  |  |  |  |
| **IGF-I** (ng/ml, N=355) |  |  |  |  |  |  |
| rs1245541 | G/A | 9.50 | (0.79,18.21) | 0.03 | 0.8 | 3.1 |
| **IGFBP-3** (ng/ml, N=355) |  |  |  |  |  |  |
| rs4234798 | T/G | 358.21 | (88.73,627.68) | 0.01 | 2.3 | 9.0 |
|  |  |  |  |  |  |  |
| **UKHLS** |  |  |  |  |  |  |
| **IGF-I** (ng/ml, N=8700)^d^ |  |  |  |  |  |  |
| rs1065656 | G/C | 0.01 | (0.001,0.02) | 0.03 | 0.02 | 1.4 |
| rs2153960 | A/G | 0.01 | (0.00,0.02) | 0.05 | 0.1 | 8.4 |
| rs4234798 | T/G | -0.01 | (-0.02,-0.004) | 0.01 | 0.1 | 5.1 |

SNPs shown are only those that were associated with serum IGFs at the conventional p-value threshold of 0.05.

^a^The effect allele increases IGF levels according to the literature.

^b^Regression models in mothers were adjusted for gestational age and 10 principal components. Regression models in children were adjusted for age at clinic visit, sex and 10 principal components. Regression models in UKHLS were adjusted for age, sex and 20 principal components.

^c^R^2^ and F-statistic from unadjusted linear regression of SNP on IGF concentration.

^d^IGF-1 concentration was natural log-transformed for analysis. Effects given correspond to the change in ln(IGF-I) per allele.

**Supplementary Table 6. Association with 15-year all-cause and prostate cancer-specific mortality of SNPs associated with IGF levels and/or with prostate cancer risk, stage or grade, in the PRACTICAL consortium.**

| **all-cause mortality** | **HR adjusted^a^** | **95% CI** | **p-value** |
| --- | --- | --- | --- |
| 13985/3396/99546^b^ |  |  |  |
| **rs11977526**^c^ |  |  |  |
| per A allele | 1.02 | (0.99,1.06) | 0.12 |
| GG | reference |  |  |
| AG | 1.04 | (0.99,1.09) | 0.09 |
| AA | 1.04 | (0.97,1.12) | 0.29 |
| p-value |  |  | 0.16 |
| **rs12666800** |  |  |  |
| per A allele | 0.99 | (0.93,1.06) | 0.87 |
| GG | reference |  |  |
| AG | 1.00 | (0.93,1.08) | 0.96 |
| AA | 0.97 | (0.73,1.28) | 0.81 |
| p-value |  |  | 0.97 |
| **rs12671457**^c,d^ |  |  |  |
| per C allele | 1.08 | (1.00,1.17) | 0.04 |
| AA | reference |  |  |
| AC | 1.11 | (1.02,1.21) | 0.01 |
| CC | 1.07 | (0.85,1.34) | 0.58 |
| p-value |  |  | 0.03 |
| **rs12702181** |  |  |  |
| per G allele | 0.95 | (0.91,1.00) | 0.03 |
| AA | reference |  |  |
| AG | 1.03 | (0.97,1.09) | 0.41 |
| GG | 0.87 | (0.77,0.99) | 0.03 |
| p-value |  |  | 0.08 |
| **rs1496499**^c^ |  |  |  |
| per G allele | 1.03 | (0.99,1.07) | 0.13 |
| TT | reference |  |  |
| GT | 1.08 | (1.05,1.12) | <0.001 |
| GG | 1.05 | (0.96,1.14) | 0.28 |
| p-value |  |  | 1x10^-4^ |
| **rs2132571**^c^ |  |  |  |
| per C allele | 1.04 | (0.99,1.09) | 0.09 |
| TT | reference |  |  |
| CT | 1.06 | (0.93,1.22) | 0.38 |
| CC | 1.09 | (0.96,1.25) | 0.18 |
| p-value |  |  | 0.21 |
| **rs2270628**^c^ |  |  |  |
| per T allele | 1.07 | (1.01,1.14) | 0.03 |
| CC | reference |  |  |
| TC | 1.07 | (1.00,1.14) | 0.07 |
| TT | 1.17 | (0.98,1.39) | 0.07 |
| p-value |  |  | 0.08 |
| **rs2453840**^c^ |  |  |  |
| per T allele | 0.97 | (0.93,1.01) | 0.18 |
| GG | reference |  |  |
| TG | 0.98 | (0.94,1.03) | 0.47 |
| TT | 0.90 | (0.78,1.04) | 0.15 |
| p-value |  |  | 0.35 |
| **rs2854744**^c^ |  |  |  |
| per A allele | 1.02 | (0.98,1.07) | 0.27 |
| CC | reference |  |  |
| AC | 1.09 | (1.04,1.14) | <0.001 |
| AA | 1.03 | (0.93,1.13) | 0.53 |
| p-value |  |  | 3x10^-4^ |
| **rs2854746**^c^ |  |  |  |
| per C allele | 1.02 | (0.97,1.06) | 0.48 |
| GG | reference |  |  |
| CG | 1.06 | (1.01,1.10) | 0.02 |
| CC | 1.01 | (0.92,1.11) | 0.84 |
| p-value |  |  | 0.02 |
| **rs2949833** |  |  |  |
| per T allele | 1.02 | (1.00,1.05) | 0.12 |
| CC | reference |  |  |
| TC | 1.05 | (1.01,1.09) | 0.01 |
| TT | 1.00 | (0.93,1.08) | 0.92 |
| p-value |  |  | 0.04 |
| **rs3110697**^c^ |  |  |  |
| per G allele | 1.00 | (0.95,1.06) | 0.94 |
| AA | reference |  |  |
| GA | 1.04 | (0.95,1.14) | 0.35 |
| GG | 1.01 | (0.90,1.14) | 0.83 |
| p-value |  |  | 0.57 |
| **rs700752**^c^ |  |  |  |
| per G allele | 0.97 | (0.92,1.02) | 0.19 |
| CC | reference |  |  |
| GC | 0.88 | (0.73,1.06) | 0.18 |
| GG | 0.89 | (0.75,1.05) | 0.18 |
| p-value |  |  | 0.41 |
| **rs924140**^c^ |  |  |  |
| per T allele | 1.02 | (0.98,1.06) | 0.26 |
| CC | reference |  |  |
| TC | 1.08 | (1.03,1.12) | <0.001 |
| TT | 1.03 | (0.95,1.12) | 0.50 |
| p-value |  |  | 0.002 |
|  |  |  |  |
| **prostate cancer mortality** |  |  |  |
| 13985/1489/99546^b^ |  |  |  |
| **rs11977526** |  |  |  |
| per A allele | 0.98 | (0.94,1.01) | 0.22 |
| GG | reference |  |  |
| AG | 1.00 | (0.95,1.06) | 0.89 |
| AA | 0.94 | (0.86,1.03) | 0.18 |
| p-value |  |  | 0.41 |
| **rs12666800**^c^ |  |  |  |
| per A allele | 0.94 | (0.85,1.03) | 0.17 |
| GG | reference |  |  |
| AG | 0.94 | (0.86,1.02) | 0.13 |
| AA | 0.87 | (0.60,1.28) | 0.49 |
| p-value |  |  | 0.31 |
| **rs12671457**^c,d^ |  |  |  |
| per C allele | 1.15 | (1.02,1.30) | 0.02 |
| AA | reference |  |  |
| AC | 1.17 | (1.05,1.30) | 0.01 |
| CC | 1.26 | (0.74,2.13) | 0.39 |
| p-value |  |  | 0.02 |
| **rs12702181** |  |  |  |
| per G allele | 0.92 | (0.88,0.96) | 0.001 |
| AA | reference |  |  |
| AG | 1.04 | (0.96,1.13) | 0.34 |
| GG | 0.76 | (0.66,0.89) | 0.001 |
| p-value |  |  | 0.002 |
| **rs1496499**^c^ |  |  |  |
| per G allele | 0.97 | (0.92,1.02) | 0.21 |
| TT | reference |  |  |
| GT | 1.03 | (0.96,1.10) | 0.42 |
| GG | 0.92 | (0.82,1.03) | 0.16 |
| p-value |  |  | 0.14 |
| **rs2132571** |  |  |  |
| per C allele | 1.01 | (0.94,1.08) | 0.80 |
| TT | reference |  |  |
| CT | 1.08 | (0.93,1.26) | 0.32 |
| CC | 1.06 | (0.89,1.25) | 0.53 |
| p-value |  |  | 0.39 |
| **rs2270628**^c^ |  |  |  |
| per T allele | 1.09 | (1.00,1.19) | 0.05 |
| CC | reference |  |  |
| TC | 1.08 | (0.96,1.22) | 0.19 |
| TT | 1.22 | (0.82,1.80) | 0.33 |
| p-value |  |  | 0.10 |
| **rs2453840**^c^ |  |  |  |
| per T allele | 0.95 | (0.91,1.00) | 0.04 |
| GG | reference |  |  |
| TG | 0.98 | (0.92,1.03) | 0.37 |
| TT | 0.83 | (0.67,1.03) | 0.09 |
| p-value |  |  | 0.12 |
| **rs2854744**^c^ |  |  |  |
| per A allele | 0.95 | (0.90,1.01) | 0.08 |
| CC | reference |  |  |
| AC | 1.04 | (0.95,1.14) | 0.37 |
| AA | 0.88 | (0.77,0.99) | 0.04 |
| p-value |  |  | 0.01 |
| **rs2854746** |  |  |  |
| per C allele | 0.96 | (0.91,1.01) | 0.12 |
| GG | reference |  |  |
| CG | 1.01 | (0.95,1.09) | 0.69 |
| CC | 0.89 | (0.78,1.00) | 0.06 |
| p-value |  |  | 0.06 |
| **rs2949833** |  |  |  |
| per T allele | 0.96 | (0.92,1.01) | 0.11 |
| CC | reference |  |  |
| TC | 1.03 | (0.99,1.08) | 0.15 |
| TT | 0.83 | (0.72,0.96) | 0.01 |
| p-value |  |  | 0.01 |
| **rs3110697**^c^ |  |  |  |
| per G allele | 0.95 | (0.89,1.01) | 0.08 |
| AA | reference |  |  |
| GA | 0.97 | (0.84,1.11) | 0.66 |
| GG | 0.90 | (0.78,1.03) | 0.12 |
| p-value |  |  | 0.15 |
| **rs700752**^c,e^ |  |  |  |
| per G allele | 0.94 | (0.91,0.98) | 0.002 |
| CC | reference |  |  |
| GC | 0.90 | (0.74,1.11) | 0.34 |
| GG | 0.87 | (0.76,1.00) | 0.06 |
| p-value |  |  | 0.004 |
| **rs924140**^c^ |  |  |  |
| per T allele | 0.96 | (0.90,1.01) | 0.10 |
| CC | reference |  |  |
| TC | 1.02 | (0.94,1.11) | 0.60 |
| TT | 0.89 | (0.80,1.00) | 0.06 |
| p-value |  |  | 0.05 |

^a^Adjusted for age and 15 principal components, and using robust standard errors to account for within-study clustering.

^b^Number of subjects/number of failures/years at risk.

^c^Non-proportional hazards (p < 0.05)

^d^In complete LD with rs12671484 (r^2^ = 1).

^e^A time-varying covariate was included in the models to allow the log hazard ratio for each of the SNPs -for which the proportional hazards assumption was not met- to change in a linear manner with time. There was evidence that the hazards ratio for rs700752 declines with time (tvc HR per G allele 0.92; 95% CI 0.85, 1.00; p = 0.05). This means that the HR for this SNP is multiplied by 0.92 whenever log time increases by 1.

**Supplementary Table 7. SNPs in the *IGFBP1/IGFBP3* region and prostate cancer risk, grade and stage in the PRACTICAL consortium.**

| **SNP** | **chr7 position^a^** | **major/minor allele** | **OR case-control^b^** | **95% CI** | **p-value** | **OR Gleason grade^b, c^** | **95% CI** | **p-value** | **OR stage^b, d^** | **95% CI** | **p-value** |
| --- | --- | --- | --- | --- | --- | --- | --- | --- | --- | --- | --- |
| rs12673958 | 45860896 | T/A | 1.00 | (0.95,1.04) | 0.94 | 0.99 | (0.94,1.04) | 0.80 | 1.00 | (0.94,1.05) | 0.89 |
| rs12666800 | 45866995 | G/A | 1.00 | (0.97,1.04) | 0.92 | 0.99 | (0.93,1.05) | 0.72 | 0.90 | (0.85,0.96) | 0.002 |
| rs12667301 | 45867304 | G/A | 1.00 | (0.95,1.04) | 0.86 | 1.01 | (0.95,1.08) | 0.77 | 1.00 | (0.93,1.06) | 0.90 |
| rs10228265 | 45869316 | A/G | 1.01 | (0.98,1.03) | 0.57 | 0.98 | (0.94,1.03) | 0.46 | 0.96 | (0.92,1.02) | 0.20 |
| rs1553009 | 45869395 | G/A | 0.98 | (0.95,1.02) | 0.37 | 1.00 | (0.96,1.04) | 0.92 | 1.02 | (0.96,1.08) | 0.57 |
| rs5002720 | 45872046 | G/A | 0.98 | (0.95,1.02) | 0.37 | 1.00 | (0.96,1.04) | 0.93 | 1.02 | (0.96,1.08) | 0.55 |
| rs2201638 | 45883311 | G/A | 0.97 | (0.90,1.04) | 0.35 | 0.96 | (0.88,1.05) | 0.40 | 0.99 | (0.92,1.08) | 0.87 |
| rs4724445 | 45883601 | G/A | 0.98 | (0.95,1.02) | 0.42 | 1.02 | (0.96,1.08) | 0.58 | 1.01 | (0.95,1.08) | 0.69 |
| rs1995051 | 45885442 | G/A | 1.00 | (0.97,1.02) | 0.86 | 0.97 | (0.94,1.00) | 0.09 | 0.99 | (0.93,1.05) | 0.71 |
| rs3763497 | 45885749 | C/T | 1.00 | (0.96,1.03) | 0.76 | 1.00 | (0.95,1.04) | 0.88 | 1.02 | (0.96,1.09) | 0.51 |
| rs1065780 | 45888078 | G/A | 1.00 | (0.97,1.02) | 0.78 | 0.99 | (0.95,1.04) | 0.81 | 1.01 | (0.96,1.06) | 0.68 |
| rs9658205 | 45889990 | T/C | 1.02 | (0.99,1.05) | 0.22 | 0.97 | (0.90,1.04) | 0.43 | 1.01 | (0.93,1.11) | 0.79 |
| rs4988515 | 45893001 | C/T | 1.00 | (0.94,1.06) | 0.89 | 0.96 | (0.89,1.03) | 0.22 | 0.95 | (0.86,1.05) | 0.31 |
| rs4619 | 45893070 | A/G | 1.00 | (0.97,1.02) | 0.83 | 1.00 | (0.95,1.05) | 0.90 | 1.01 | (0.94,1.08) | 0.80 |
| rs1908751 | 45895920 | C/T | 0.99 | (0.96,1.01) | 0.41 | 0.96 | (0.93,1.00) | 0.03 | 1.00 | (0.93,1.06) | 0.99 |
| rs10224141 | 45898425 | A/C | 0.98 | (0.93,1.02) | 0.29 | 1.03 | (0.97,1.09) | 0.29 | 1.01 | (0.95,1.06) | 0.79 |
| rs1496496 | 45901304 | A/G | 1.00 | (0.97,1.02) | 0.79 | 1.00 | (0.95,1.05) | 0.88 | 1.01 | (0.94,1.08) | 0.85 |
| rs1496497 | 45901444 | T/G | 1.02 | (0.99,1.04) | 0.21 | 0.98 | (0.93,1.03) | 0.41 | 1.00 | (0.93,1.08) | 0.89 |
| rs12702181 | 45905870 | A/G | 0.99 | (0.96,1.02) | 0.41 | 0.95 | (0.92,0.99) | 0.01 | 0.99 | (0.93,1.05) | 0.82 |
| rs12671457 | 45907327 | A/C | 1.03 | (1.01,1.06) | 0.02 | 0.98 | (0.92,1.05) | 0.56 | 1.02 | (0.94,1.11) | 0.57 |
| rs12671484 | 45907556 | A/G | 1.03 | (1.01,1.06) | 0.02 | 0.98 | (0.92,1.05) | 0.55 | 1.02 | (0.94,1.11) | 0.56 |
| rs2270628 | 45909971 | C/T | 1.03 | (1.00,1.05) | 0.03 | 0.97 | (0.93,1.02) | 0.28 | 1.01 | (0.94,1.08) | 0.86 |
| rs10282088 | 45910847 | C/A | 0.98 | (0.93,1.02) | 0.32 | 1.03 | (0.97,1.09) | 0.27 | 1.01 | (0.95,1.08) | 0.74 |
| rs13223993 | 45911631 | G/A | 0.98 | (0.94,1.02) | 0.40 | 1.01 | (0.96,1.06) | 0.67 | 0.99 | (0.93,1.04) | 0.71 |
| rs6670 | 45912655 | T/A | 1.01 | (0.98,1.05) | 0.51 | 1.00 | (0.94,1.05) | 0.87 | 1.03 | (0.97,1.10) | 0.29 |
| rs2453840 | 45914213 | G/T | 0.97 | (0.95,0.99) | 0.01 | 0.98 | (0.94,1.02) | 0.30 | 1.00 | (0.94,1.05) | 0.97 |
| rs10255707 | 45915093 | C/T | 0.98 | (0.95,1.02) | 0.31 | 0.96 | (0.89,1.03) | 0.27 | 0.96 | (0.88,1.05) | 0.38 |
| rs6953668 | 45916276 | G/A | 0.84 | (0.70,1.01) | 0.07 | 1.14 | (0.65,1.96) | 0.66 | 0.85 | (0.63,1.15) | 0.29 |
| rs2471551 | 45917456 | G/C | 1.01 | (0.96,1.05) | 0.73 | 0.98 | (0.93,1.02) | 0.34 | 0.99 | (0.93,1.04) | 0.64 |
| rs3793345 | 45918079 | T/C | 1.01 | (0.97,1.05) | 0.72 | 0.97 | (0.93,1.02) | 0.23 | 0.98 | (0.93,1.03) | 0.44 |
| rs2854747 | 45920318 | G/A | 1.00 | (0.99,1.02) | 0.65 | 1.04 | (0.99,1.09) | 0.15 | 1.04 | (0.97,1.11) | 0.31 |
| rs2132572 | 45921946 | C/T | 0.98 | (0.95,1.01) | 0.21 | 0.97 | (0.92,1.03) | 0.37 | 0.96 | (0.88,1.04) | 0.32 |
| rs2132570 | 45922864 | C/A | 0.98 | (0.95,1.01) | 0.20 | 0.97 | (0.91,1.03) | 0.35 | 0.96 | (0.88,1.04) | 0.32 |
| rs2453836 | 45926196 | A/G | 1.02 | (0.98,1.06) | 0.36 | 0.97 | (0.93,1.03) | 0.34 | 0.99 | (0.93,1.04) | 0.67 |
| rs2960436 | 45937683 | G/A | 1.02 | (1.00,1.04) | 0.07 | 1.04 | (1.00,1.10) | 0.07 | 1.03 | (0.96,1.11) | 0.34 |
| rs1534151 | 45945046 | A/G | 1.00 | (0.96,1.03) | 0.83 | 0.97 | (0.93,1.01) | 0.17 | 0.99 | (0.95,1.02) | 0.49 |
| rs2949833 | 45945221 | C/T | 1.01 | (0.99,1.04) | 0.22 | 1.08 | (1.02,1.12) | 0.002 | 1.04 | (0.96,1.12) | 0.33 |
| rs1534150 | 45945691 | T/C | 1.00 | (0.96,1.03) | 0.83 | 0.97 | (0.93,1.01) | 0.17 | 0.99 | (0.95,1.02) | 0.50 |
| rs788718 | 45966945 | G/A | 1.02 | (0.98,1.06) | 0.35 | 0.96 | (0.91,1.02) | 0.22 | 0.96 | (0.92,1.01) | 0.13 |

^a^GRCh38.p2.

^b^Effect of minor allele is shown. Regression models adjusted for age and 15 principal components, using robust standard errors to account for within-study clustering.

^c^Gleason grade: <7 (reference) vs ≥7.

^d^Stage: localised (reference) vs advanced.

22,939 cases/19,996 controls.

9,429 low grade (<7)/8,913 high grade (≥7) disease.

14,235 localised/4,455 advanced disease.

**Supplementary Table 8. Non-*IGFBP-1/IGFBP3* SNPs from the discovery GWAS**^2^ **and prostate cancer risk, grade and stage in the PRACTICAL consortium.**

| **SNP** | **chromosomal position^a^** | **effect/non-effect allele^b^** | **OR case-control^c^** | **95% CI** | **p-value** | **OR Gleason grade^c, d^** | **95% CI** | **p-value** | **OR stage^c, e^** | **95% CI** | **p-value** |
| --- | --- | --- | --- | --- | --- | --- | --- | --- | --- | --- | --- |
| rs4234798 | 4:7218206 | T/G | 1.00 | (0.98,1.03) | 0.87 | 0.99 | (0.95,1.03) | 0.56 | 1.04 | (0.99,1.09) | 0.15 |
| rs2153960 | 6:108666981 | A/G | 1.00 | (0.98,1.02) | 0.98 | 1.05 | (1.01,1.11) | 0.03 | 0.93 | (0.89,0.98) | 0.01 |
| rs7780564 | 7:7843692 | C/A | 1.00 | (0.96,1.03) | 0.82 | 1.01 | (0.94,1.08) | 0.80 | 1.03 | (0.97,1.09) | 0.35 |
| rs1245541 | 10:72089881 | G/A | 1.00 | (0.97,1.03) | 0.98 | 1.05 | (1.00,1.11) | 0.07 | 0.97 | (0.89,1.05) | 0.39 |
| rs1065656 | 16:1788835 | G/C | 1.02 | (0.99,1.05) | 0.15 | 1.01 | (0.96,1.06) | 0.73 | 1.02 | (0.95,1.10) | 0.57 |

^a^GRCh38.p2.

^b^The effect allele increases IGF levels according to the discovery GWAS^2^.

^c^Regression models adjusted for age and 15 principal components, using robust standard errors to account for within-study clustering.

^d^Gleason grade: <7 (reference) vs ≥7.

^e^Stage: localised (reference) vs advanced.

22,939 cases/19,996 controls.

9,429 low grade (<7)/8,913 high grade (≥7) disease.

14,235 localised/4,455 advanced disease.

**Supplementary Figure 1. Pattern of linkage disequilibrium (LD) in the *IGFBP1/IGFBP3* gene region.**

47 SNPs are depicted, those validated as instrumental variables for IGF levels and those with the strongest associations with prostate cancer are shown.

Red dots: SNPs associated with prostate cancer risk. Green dots: SNPs associated with prostate cancer grade. Blue dot: SNP associated with prostate cancer stage. Orange dots: SNPs associated with all-cause and prostate cancer-specific mortality.

LD r^2^ values were obtained using LDlink and selecting the GBR population (English and Scottish). The LD plot was created with LDheatmap in R.

SNPs in the diagram are evenly separated for the sake of clarity. The majority of SNPs lie between 45,861 (rs12673958) and 45,969 kbs (rs11977526) whereas rs700752 is located at 46,714 kbs (GRCh38.p2).

**
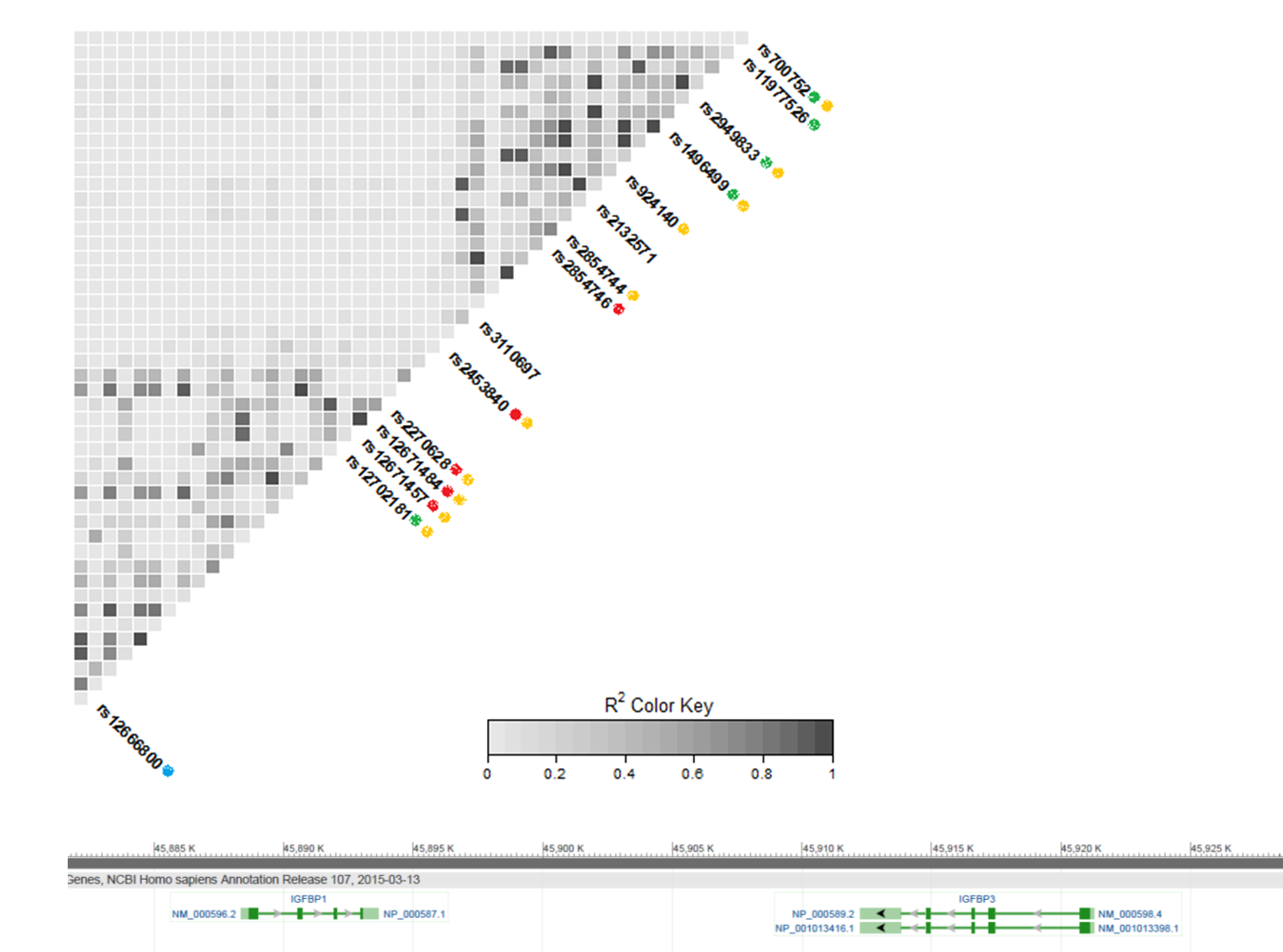
**

**Supplementary Figure 2. Fixed-effects meta-analysis of the association of SNP rs11977526 with Gleason grade in the PRACTICAL consortium.**


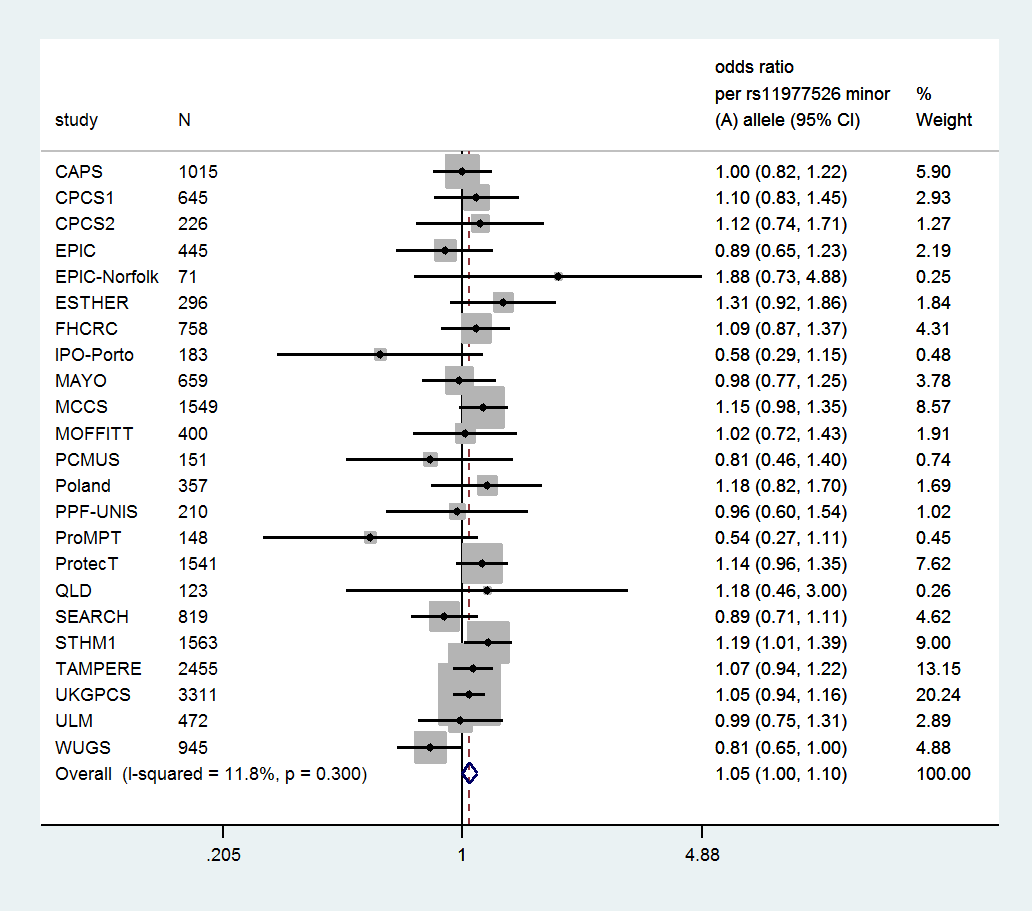


Adjusted for age and 15 principal components.

**Supplementary Figure 3. Fixed-effects meta-analysis of the association of SNP rs700752 with Gleason grade in the PRACTICAL consortium.**


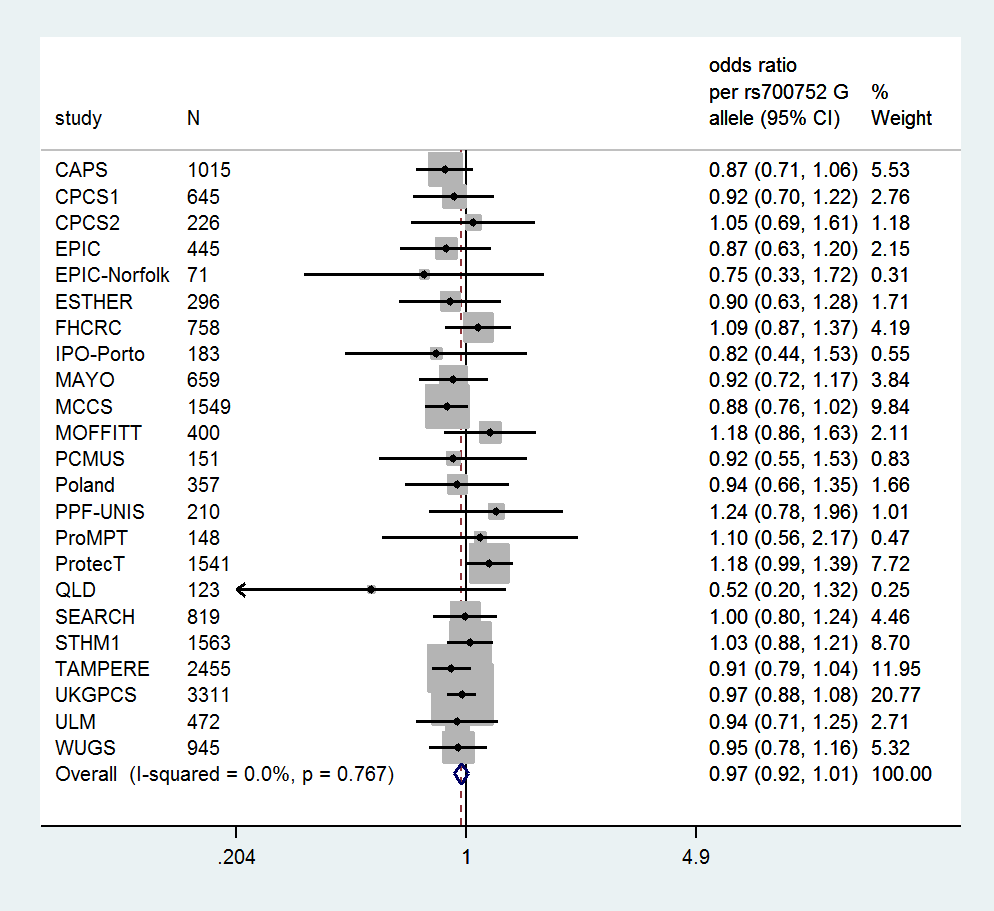


Adjusted for age and 15 principal components.

****The PRACTICAL CONSORTIUM (in addition to those named in the author list)**

Additional members from the consortium are: Margaret Cook^1^, Angela Morga^2^, Artitaya Lophatananon^3,4^, Cyril Fisher^2^, Daniel Leongamornlert^2^, Edward J. Saunders^2^, Emma J. Sawyer^2^, Koveela Govindasami^2^, Malgorzata Tymrakiewicz^2^, Michelle Guy^2^, Naomi Livni^2^, Rosemary Wilkinson^2^, Sara Jugurnauth-Little^2^, Steve Hazel^2^, Tokhir Dadaev^2^, Melissa C. Southey^5^, Liesel M. Fitzgerald^6^, Robert MacInnis^6,7^, John Pedersen^8^, John Hopper^8^, Ami Karlsson^9^, Carin Cavalli-Bjoerkman^9^, Jan-Erik Johansson^9^, Jan Adolfson^9^, Markus Aly^9,10^, Michael Broms^9^, Paer Stattin^9^, Brian E. Henderson^11^, Fredrick Schumacher^11^, Anssi Auvinen^12^, Kimmo Taari^13^, Liisa Maeaettaenen^14^, Paula Kujala^15^, Teemu Murtola^16,17^, Teuvo LJ Tammela^17^, Csilla Sipeky^18^, Martin Andreas Roder^19^, Peter Iversen^19^, Peter Klarskov^20^, Sune F. Nielsen^21,22^, Maren Weischer^21,22^, Tim J. Key^23^, Hans Wallinder^24^, Sven Gustafsson^24^, Angela Cox^25^, Anne George^26^, Athene Lane^27^, Gemma Marsden^28^, Michael Davis^27^, Paul Brown^27^, Paul Pharoah^29^, Lisa B. Signorello^31,30^, Wei Zheng^32^, Shannon K. McDonnell^33^, Daniel J. Schaid^33^, Liang Wang^33^, Lori Tillmans^33^, Shaun Riska^33^, Thomas Schnoeller^34^, Kathleen Herkommer^35^, Manuel Luedeke^34^, Walther Vogel^36^, Dominika Wokozorczyk^37^, Jan Lubiski^37^, Wojciech Kluzniak^37^, Katja Butterbach^38^, Christa Stegmaier^39^, Bernd Holleczek^39^, Babu Zachariah^40^, Hui-Yi Lim^41^, Hyun Park^40^, James Haley^40^, Julio Pow-Sang^40^, Maria Rincon^40^, Selina Radlein^40^, Thomas Sellers^40^, Chavdar Slavov^42^, Aleksandrina Vlahova^43^, Atanaska Mitkova^44^, Darina Kachakova^44^, Elenko Popov^42^, Svetlana Christova^43^, Tihomir Dikov^43^, Vanio Mitev^44^, Allison Eckert^45^, Angus Collins^45^, Glenn Wood^45^, Greg Malone^45^, Judith A. Clements^45,46^, Kris Kerr^45^, Megan Turner^45^, Pamela Saunders^45^, Peter Heathcote^45^, Gail Risbridger^45^, Wayne Tilley^45^, Lisa Horvath^45^, Trina Yeadon^45^, Srilakshmi Srinivasan^46^, Leire Moya^46^, Amanda Spurdle^47^, Joana Santos^48^, Carmen Jerónimo^48^, Paula Paulo^48^, Pedro Pinto^48^, Rui Henrique^48^, Sofia Maia^48^, Agnieszka Michael^49^, Andrzej Kierzek^49^, Huihai Wu^49^

^1^Centre for Cancer Genetic Epidemiology, Department of Public Health and Primary Care, University of Cambridge, Strangeways Laboratory, Worts Causeway, Cambridge CB1 8RN, UK, ^2^The Institute of Cancer Research, Sutton, UK, ^3^Institute of Population Health, University of Manchester, Manchester, UK, ^4^Warwick Medical School, University of Warwick, Coventry, UK, ^5^Genetic Epidemiology Laboratory, Department of Pathology, The University of Melbourne, Grattan Street, Parkville, Victoria 3010, Australia, ^6^Cancer Epidemiology Centre, The Cancer Council Victoria, 615 St Kilda Road, Melbourne, Victoria, Australia, Australia, ^7^Centre for Epidemiology and Biostatistics, Melbourne School of Population and Global Health, The University of Melbourne, Victoria, Australia, ^8^Tissupath Pty Ltd., Melbourne,Victoria 3122, ^9^Department of Medical Epidemiology and Biostatistics, Karolinska Institute, Stockholm, Sweden, ^10^Department of Clinical Sciences at Danderyds Hospital, Stockholm, Sweden, ^11^Department of Preventive Medicine, Keck School of Medicine, University of Southern California/Norris Comprehensive Cancer Center, Los Angeles, California, USA, ^12^Department of Epidemiology, School of Health Sciences, University of Tampere, Tampere, Finland, ^13^Department of Urology, Helsinki University Central Hospital and University of Helsinki, Helsinki, Finland, ^14^Finnish Cancer Registry, Helsinki, Finland, ^15^Fimlab Laboratories, Tampere University Hospital, Tampere, Finland, ^16^School of Medicine, University of Tampere, Tampere, Finland, ^17^Department of Urology, Tampere University Hospital and Medical School, University of Tampere, Finland, ^18^Department of Medical Biochemistry and Genetics, Institute of Biomedicine, University of Turku, Turku, Finland, ^19^Copenhagen Prostate Cancer Center, Department of Urology, Rigshospitalet, Copenhagen University Hospital, Tagensvej 20, 7521, DK-2200 Copenhagen, Denmark, ^20^Department of Urology, Herlev and Gentofte Hospital, Copenhagen University Hospital, Herlev Ringvej 75, DK-2730 Herlev, Denmark, ^21^Department of Clinical Biochemistry, Herlev Hospital, Copenhagen University Hospital, Herlev Ringvej 75, DK-230 Herlev, Denmark, ^22^Faculty of Health and Medical Sciences, University of Copenhagen, ^23^Cancer Epidemiology Unit, Nuffield Department of Clinical Medicine, University of Oxford, Oxford, UK, ^24^Department of Epidemiology and Biostatistics, School of Public Health, Imperial College, London, UK, ^25^CR-UK/YCR Sheffield Cancer Research Centre, University of Sheffield, Sheffield, UK, ^26^University of Cambridge, Department of Oncology, Box 279, Addenbrooke's Hospital, Hills Road Cambridge CB2 0QQ, UK, ^27^School of Social and Community Medicine, University of Bristol, Canynge Hall, 39 Whatley Road, Bristol, BS8 2PS, UK, ^28^Nuffield Department of Surgical Sciences, University of Oxford, Oxford, UK, Faculty of Medical Science, University of Oxford, John Radcliffe Hospital, Oxford, UK, ^29^Centre for Cancer Genetic Epidemiology, Department of Oncology, University of Cambridge, Strangeways Laboratory, Worts Causeway, Cambridge, UK, ^30^International Epidemiology Institute, 1555 Research Blvd., Suite 550, Rockville, MD 20850, USA, ^31^Department of Epidemiology, Harvard School of Public Health, 677 Huntington Avenue, Boston, MA 02115, USA, ^32^Division of Epidemiology, Department of Medicine, Vanderbilt University Medical Center, 2525 West End Avenue, Suite 800, Nashville, TN 37232 USA, ^33^Mayo Clinic, Rochester, Minnesota, USA, ^34^Department of Urology, University Hospital Ulm, Germany, ^35^Department of Urology, Klinikum rechts der Isar der Technischen Universitaet Muenchen, Munich, Germany, ^36^Institute of Human Genetics, University Hospital Ulm, Germany, ^37^International Hereditary Cancer Center, Department of Genetics and Pathology, Pomeranian Medical University, Szczecin, Poland, ^38^Division of Clinical Epidemiology and Aging Research, German Cancer Research Center (DKFZ), 69120 Heidelberg, Germany, ^39^Saarland Cancer Registry, 66119 Saarbruecken, Germany, ^40^Department of Cancer Epidemiology, Moffitt Cancer Center, 12902 Magnolia Drive, Tampa, FL 33612, USA, ^41^Biostatistics Program, Moffitt Cancer Center, 12902 Magnolia Drive, Tampa, FL 33612, USA, ^42^Department of Urology and Alexandrovska University Hospital, Medical University, Sofia, Bulgaria, ^43^Department of General and Clinical Pathology, Medical University, Sofia, Bulgaria, ^44^Department of Medical Chemistry and Biochemistry, Molecular Medicine Center, Medical University, Sofia, 2 Zdrave Str., 1431 Sofia, Bulgaria, ^45^Australian Prostate Cancer BioResource, Brisbane, Australia, ^46^Australian Prostate Cancer Research Centre-Qld, Institute of Health and Biomedical Innovation and School of Biomedical Science, Queensland University of Technology, Brisbane, Australia, ^47^Molecular Cancer Epidemiology Laboratory, Queensland Institute of Medical Research, Brisbane, Australia, ^48^Department of Genetics, Portuguese Oncology Institute, Porto, Portugal, ^49^The University of Surrey, Guildford, Surrey GU2 7XH, UK.
